# Dynamic Rendering of the Heterogeneous Cell Response to Anticancer Treatments

**DOI:** 10.1371/journal.pcbi.1003293

**Published:** 2013-10-17

**Authors:** Francesca Falcetta, Monica Lupi, Valentina Colombo, Paolo Ubezio

**Affiliations:** Biophysics Unit, Laboratory of Anticancer Pharmacology, Department of Oncology, IRCCS - Istituto di Ricerche Farmacologiche “Mario Negri,” Milano, Italy; National University of Singapore, Singapore

## Abstract

The antiproliferative response to anticancer treatment is the result of concurrent responses in all cell cycle phases, extending over several cell generations, whose complexity is not captured by current methods. In the proposed experimental/computational approach, the contemporary use of time-lapse live cell microscopy and flow cytometric data supported the computer rendering of the proliferative process through the cell cycle and subsequent generations during/after treatment. The effects of treatments were modelled with modules describing the functional activity of the main pathways causing arrest, repair and cell death in each phase. A framework modelling environment was created, enabling us to apply different types of modules in each phase and test models at the complexity level justified by the available data. We challenged the method with time-course measures taken in parallel with flow cytometry and time-lapse live cell microscopy in X-ray-treated human ovarian cancer cells, spanning a wide range of doses. The most suitable model of the treatment, including the dose-response of each effect, was progressively built, combining modules with a rational strategy and fitting simultaneously all data of different doses and platforms. The final model gave for the first time the complete rendering *in silico* of the cycling process following X-ray exposure, providing separate and quantitative measures of the dose-dependence of G_1_, S and G_2_M checkpoint activities in subsequent generations, reconciling known effects of ionizing radiations and new insights in a unique scenario.

## Author Summary

The antiproliferative response to anticancer treatment is the result of concurrent effects in all cell cycle phases, where molecular control pathways (checkpoints) are activated and cells may be arrested to repair DNA damage or killed if not able to succeed in the repair process. The complexity and inter-cell variability of these phenomena are not captured by the available methods, and the origin of the dose-dependence of the response remains elusive. In this work, we present an experimental-computational method that discloses and measures the individual responses of cell cycle controls in each phase and generation. We demonstrate that the method, exploiting jointly data sets obtained by flow cytometry and time-lapse *in vivo* imaging with a suitable experimental design, is able to achieve a full reconstruction *in silico* of the actual movement of cell cohorts following X-ray exposure, providing separate and quantitative measures of the dose-dependence of G_1_, S and G_2_M checkpoint activities in subsequent generations. Best fit parameters values are actual measures of the probability of activation of the specific pathways of arrest, repair or death within the cell population, linking the molecular scale to the “macroscopic” response, with full appreciation of its dynamics and inter-cell heterogeneity.

## Introduction

Anticancer research spans a wide range of scales, from the microscopic/molecular up to the macroscopic level of clinical assessment of treatment efficacy. On an intermediate scale of preclinical testing *in vitro* and *in vivo*, cancer research maintains the need to evaluate the antiproliferative activity of old and new treatment options in cancer cell populations. In fact, even for new drugs, assessment of activity against a molecular target does not guarantee antiproliferative activity and control of expansion – if not eradication – of cancer cells, which is clearly the primary goal of any treatment. However, the impressive advances in the molecular understanding of cancer(s) have not been accompanied by a deeper comprehension of proliferation in treated samples, often simplistically estimated with scores based on treated/control ratios. These do not catch the complexity of the phenomena in play, leaving unanswered several questions, from the contribution of inter-cell heterogeneity to the origin of dose-dependence, from the dynamics of the response of checkpoint controls to the overlapping of cytostatic and cytotoxic phenomena [1]–[3]. In particular, biomedical research is now acknowledging the importance of cell-to-cell diversity and dynamic variability for protein levels [4]–[7] and response to environmental interactions [8] or treatments [9], even within populations of genetically identical cells. This calls for a probabilistic view of the single cell response representing a change of perspective compared to exclusively molecularly-driven research, often dominated by a deterministic-mechanistic view according to which a drug makes a given effect or not, without specifying the probabilities of the events and their dose-dependence.

Mathematical modelling is increasingly adopted to tackle biological complexity, together with experimental procedures producing appropriate quantitative data, for *in silico* rendering of biological structures and processes in different fields and scales, from X-ray crystallography to medical imaging [10]–. The question is normally tackled by adopting a computational model of the biological phenomenon, whose inputs are meaningful biological parameters and outputs are measurable quantities. For instance, in the crystallography field a model of the diffraction keeps the 3D structure of a molecule as input and gives as output the data that a molecule's crystal would produce when challenged in X-ray diffraction experiments. The model can be used in two ways: to infer the 3D structure from experimental data (optimization problem) or to simulate the expected data from hypothetical 3D structures (simulation) [15].

**Figure 1:**
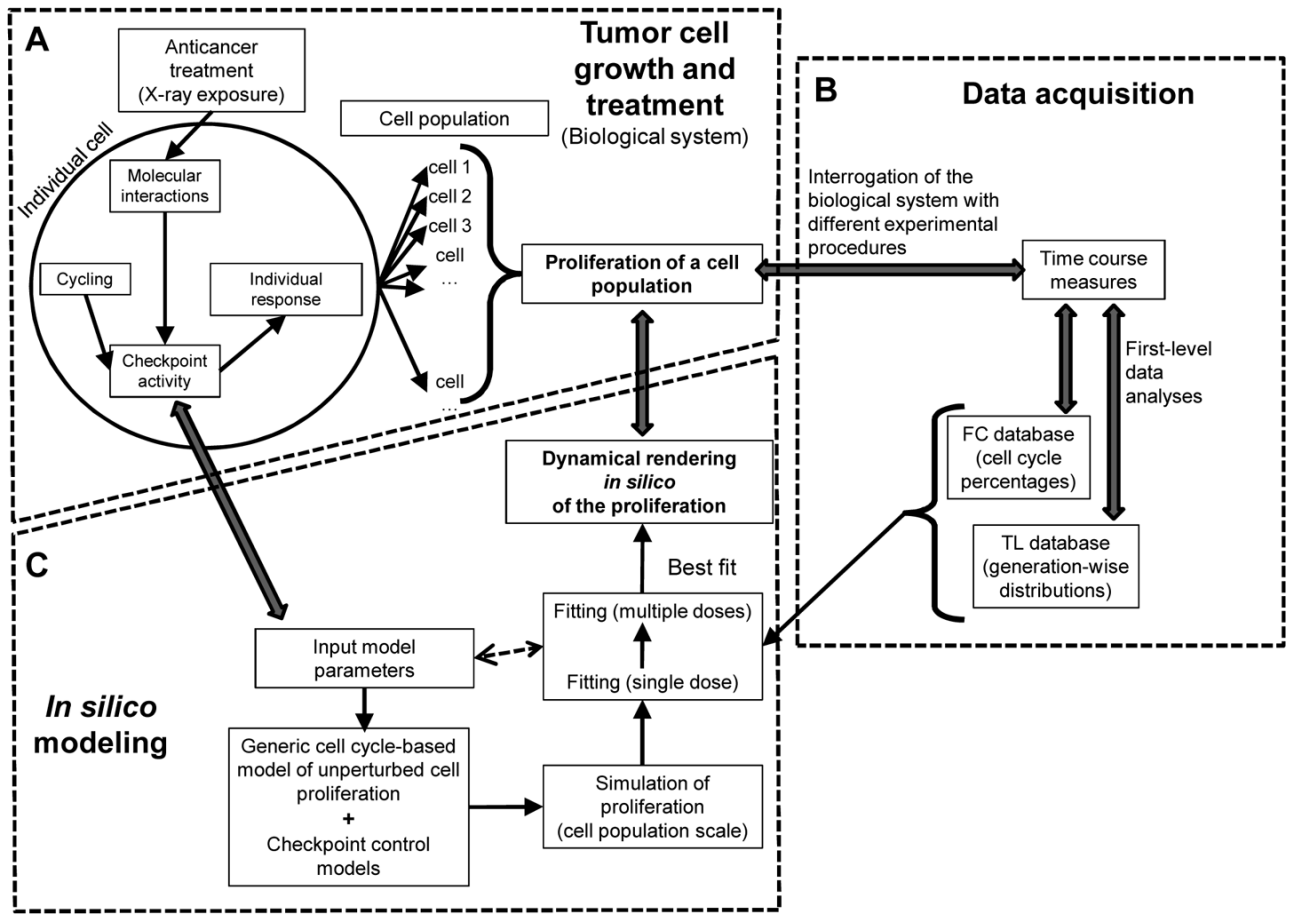
Outline of the experimental/computational method. (A) Individual cancer cells respond to the challenge of a treatment activating molecular pathways that cause cell cycle arrest, damage repair or cell death. The response has complex time-dependence and the effects are still detectable in the descendants of the cells exposed to the drugs. Cell outcomes are not homogeneous, and the overall antiproliferative response at the cell population level is the sum of the different stories of all cells. (B) Different experimental techniques can be applied to interrogate the biological system and retrieve information about cell proliferation during or after treatment. Flow cytometry gives percentages of cells in the various cycle phases, while time-lapse live cell microscopy indicates the propagation of the effects through subsequent cell generations. A proper experimental plan, including time course measures with both techniques on samples treated with different doses, can potentially give a complete scenario of the effects in play, but is not easy to interpret. (C) A computer model renders *in silico* the dynamics of cell proliferation, based on parameters associated with unperturbed growth and the activity of cell cycle checkpoints, producing outputs that mimic experimental data obtained with both FC and TL platforms. A best fit rendering fully consistent with all experimental data discloses the details of the proliferation and of the underlying checkpoint activities, with their dose-dependence.

Adopting a conceptually similar approach, we present here a mixed experimental/computational method (Figure 1) to render the process of proliferation at the cell population level, using a computational model whose input parameters are simple descriptors of the functional activities of the main intracellular molecular controls of the cell cycle and whose outputs can be directly fitted to data obtained by time-lapse live cell microscopy (TL) and DNA flow cytometry (FC). The two platforms convey complementary information, FC focusing on cell distributions in G_1_, S, G_2_M cell cycle phases, TL on lineage trees following cells in subsequent generations.

Valuable examples of modelling proliferation over subsequent generations can be found in the immunological field, were data from cells labelled with suitable trackers are exploited to follow lymphocyte expansion after introduction of stimuli triggering their entry in cycle [16], [17]. These models usually do not include cell cycle phases. On the other hand, models based on cell cycle phases were used [18], [19] and adapted to model treatment [20] not distinguishing generations, although effects of treatment were observed not only in cells directly exposed but also to their descendants [21], [22].

In this paper we attempt to build a model deciphering the effects of treatment including both cell cycle phases and subsequent generations, exploiting together FC and TL data. By modelling proliferation at the cell population level it was possible to incorporate different kinds of heterogeneity in the structure of the model, both for untreated and treated cell populations. For untreated cells, intercell heterogeneity was included by distinguishing G_1_, S and G_2_M phases and introducing frequency distributions of the duration of each phase. After treatment, observations demonstrate that some cells die other survive, some are blocked in a given phase, and among them some die and others cycle and reach subsequent generations and so on. In this case the heterogeneity is rendered by the model parameters themselves, which are probabilities or rates of occurrence of each phenomenon in play and were applied to the cell cohorts arriving at each checkpoint at a given time.

A multi-phase and multi-generation model for proliferation in the absence of treatment was already presented [23] showing the feasibility to reproduce proliferation at a deeper level than models explaining simply the time course of the overall cell number or equivalent measures, or cell phase percentages alone or cell generations alone. This model was here modified to include the effects of a treatment. Our previous attempts in treatment modelling were based on “single-cycle” models, where cells after division re-enter in the same cycle and generations were mixed, and on FC data, alone or coupled with absolute cell counts, with simplified descriptions of the time-dependence of checkpoint activities, and trial-and-error simulations [24]. These treatment models were revised and modules rendering the main effects of treatments were included in each phase and generation of the multi-phase and multi-generation model. This eventually enabled us to explain most of time-dependences required in the previous treatment models as different effects in subsequent generations. Moreover, a non-linear fitting procedure was included and, correspondingly, the experimental design was strongly enriched, with the inclusion of TL data, for different treatment doses.

Setting the method obliged us to study and solve a number of related subproblems, concerning the data structure, the cell cycle model and the optimization (Figure 1). A robust rendering of the proliferation process up to the fourth generation was achieved, designing a formal workflow for model building and fitting, considering contemporaneously data from treatments at different doses and exploiting cross-validation between FC and TL datasets.

This approach was used here to disclose the details of the proliferation of a cancer cell population after X-ray exposure, leading to a measure of the dose- and time-dependence of the main checkpoint activities and of the balance between recycling and death over three cell generations after treatment. Reconstruction of the process was then used for *in silico* experiments, evaluating the consequences of default or potentiation of specific checkpoints on the overall effect of treatment.

## Results

### Experimental plan and data structure

**Figure 2:**
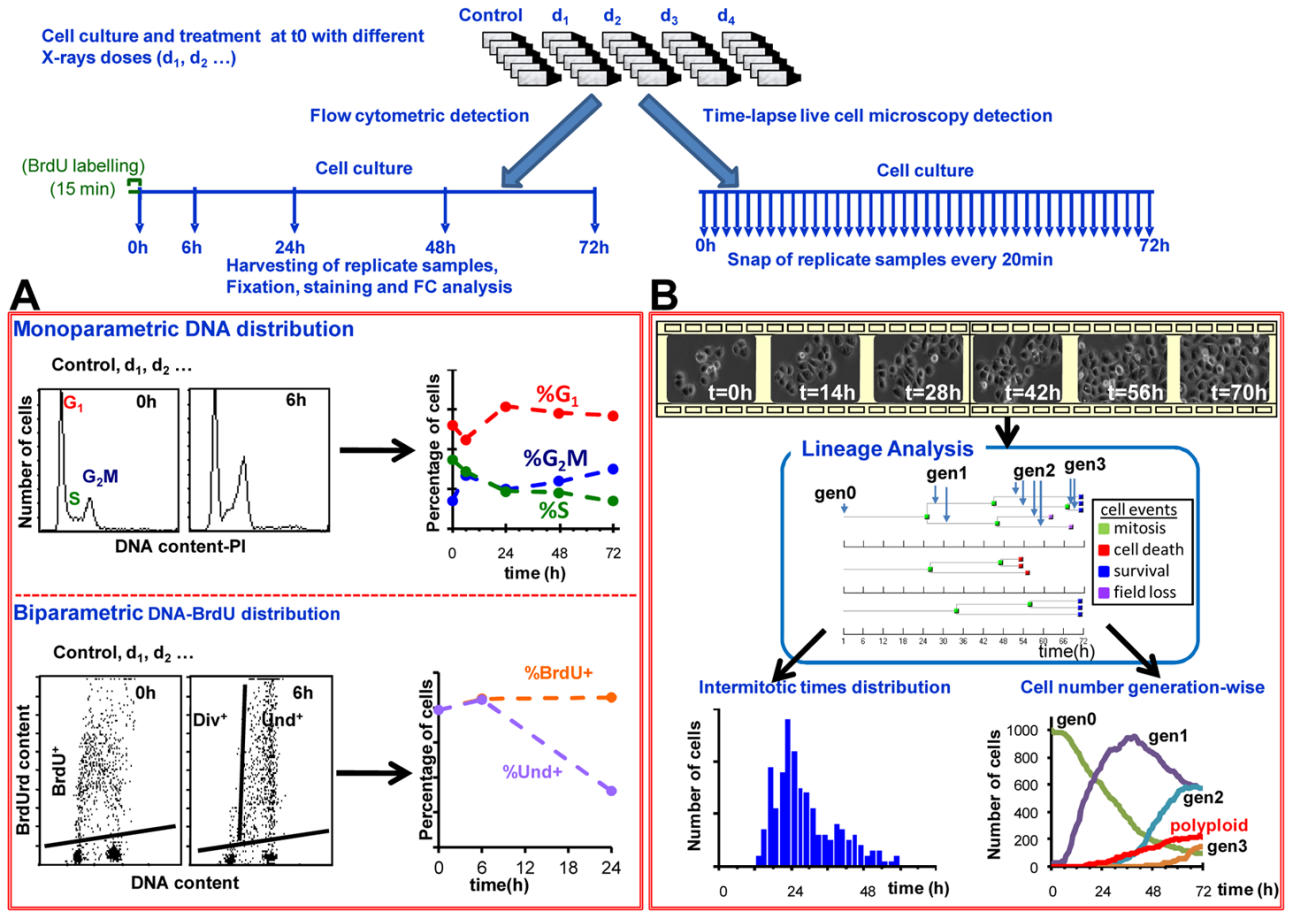
Experimental plan and first-level data analysis. For FC analysis, cells from replicated samples were collected, fixed and stained at 6, 24, 48 and 72(A) Representative FC data: i) monoparametric cell cycle analysis, from which we calculated %G_1_, %S, %G_2_M, ii) biparametric DNA-BrdU analysis after BrdU pulse labeling, from which we calculated the percentages of BrdU-positive cells (%BrdU^+^), i.e. cells which were in S phase at the time of labelling, and undivided BrdU^+^ cells (%Und^+^). (B) Representative TL data: movies were collected and each cell was tracked with its descendants. At least 300 lineages were analysed in each treatment group. Three representative lineages are shown, where coloured squares indicate the outcome event of each cell. Analysis of lineage data gave population statistics like frequency distribution of intermitotic times and number of cells in each generation (bottom).

Human ovarian cancer cells (IGROV-1) in exponential growth were exposed to different doses of X-rays (0 h). The response to the treatment was monitored over time using TL and FC (Figure 2). Two FC techniques were applied (Figure 2A): i) monoparametric cell cycle analysis, which gave FC DNA histograms, ii) BrdU pulse-chase, which gave biparametric DNA-BrdU histograms at different times after treatment. Through a first-level analysis (supplementary Text S1), we obtained time-courses of FC cell percentages in cell cycle phases and BrdU subsets, which were collected in the FC database.

TL movies (see representative examples as supplementary Videos 1 and 2) were analysed tracking all the cells in the fields of view at 0 h, defined as “generation 0” (gen0), and their descendants, constituting gen1, gen2 etc. Observations were collected in a “lineage database” reporting for each cell: i) a cell identification code, including information about treatment, field, lineage and generation number; ii) time of birth; iii) time and kind of outcome (mitosis (M), death (D), survival at 72 h (S), re-fusion of two newborn cells to make a polyploid one (R) or loss from the field of view (FL)) (Figure 2B). Other events, like anomalous mitoses (resulting in three or four offspring), occurred with a frequency lower than 1% and were neglected in this analysis.

Lineage data were then analysed, deriving cell population statistics. For each dose we calculated: i) the frequency distribution of intermitotic times; ii) the time course of the number of cells in each generation and of polyploid cells, relative to the number of cells present at 0 h and corrected for FL cells; iii) the frequency of the cell events in each generation. These data made up the three sections of the TL database.

Qualitative visual inspection of FC and TL data after this first level of analysis already enabled us to depict a preliminary view of the effects of X-ray exposure (supplementary Text S2). TL data (Figure S1) indicated the presence of cell cycle delays even at 0.5 Gy, and cell death was detectable from 2.5 Gy, and up to gen2; some sibling cells eventually re-fused, producing a subset of polyploid cells within which mitosis was a rare event. FC data provided complementary information, indicating the activation of blocks/delays in all cell cycle phases, but without distinguishing different generations (Figures S2, S3).

### Cell cycling models

**Figure 3:**
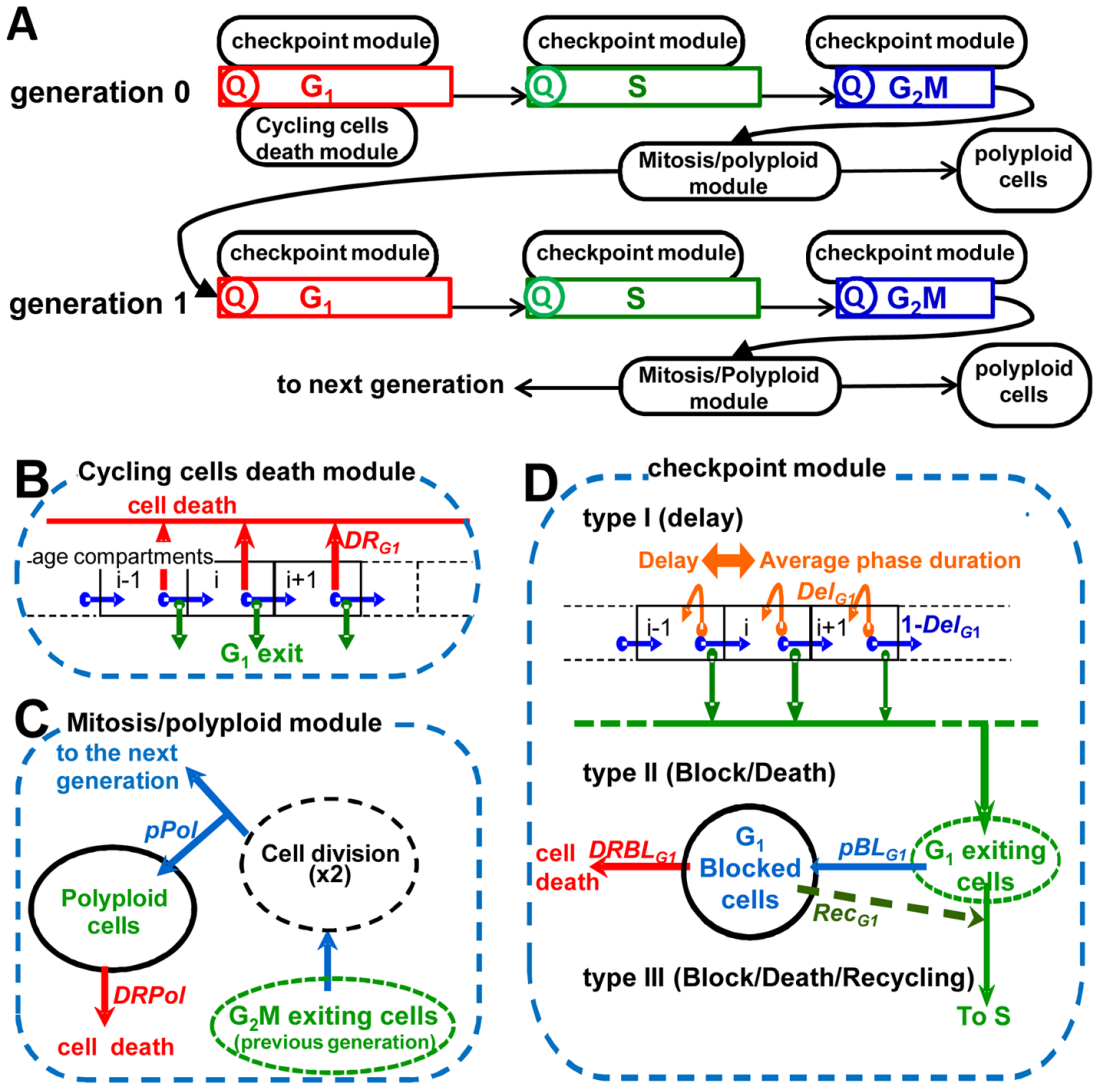
Structure of the modelling framework, with detail of cell progression and perturbation modules. (A) The model reproduces the flow of cell cohorts through the cell cycle phases and subsequent generations, each phase including specific quiescence (Q) and perturbation modules. A cohort of cells entering a phase is first processed by the quiescence module, committing a fraction of them to the G_1_ quiescence compartment. Then untreated cells progress through the subsequent age compartments of each phase, exiting at different ages as shown in Figure S4 and detailed in supplementary Text S3. In treated samples this process is altered by perturbation modules, which can be applied to any phase, providing a flexible framework to build proliferation models with the desired complexity. (B) Cycling cell death module, exemplified for G_1_ phase. It applies first-order death kinetics to all cycling cells in phase G_1_ with a rate *DR_G1_*. (C) The mitosis/polyploid module acts on dividing cells, doubling the number of cells exiting G_2_M and assuming that a fraction (*pPol*) of newborn siblings re-fuse, collecting re-fused (polyploid) cells in a separate compartment, from which they die with a rate *DRPol*. (D) Checkpoint modules, exemplified for G_1_ phase. A specific type of checkpoint module can be selected, as described in Computational Methods, with parameters *Del_G1_* (producing a transit delay), *pBL_G1_* (block probability), *DRBL_G1_* (death rate of blocked cells), *Rec_G1_* (recycling rate of blocked cells).

The overall process of proliferation of cancer cell populations involves the passage of cell cohorts through cycle phases in subsequent generations. Treatment changes the cycling (i.e. the “unperturbed” cycling of untreated controls), imposing delays and blocks and killing cells with different mechanisms in each phase. This is reflected in the structure of the modelling framework implemented *in silico* (Figure 3A). Response to treatment was modelled with modules for G_1_, S and G_2_M checkpoint response in gen0, gen1, gen2 etc. superimposing and modifying the flow of the cells through the cycle. An additional module governs polyploidization at the end of each generation. The user interface of the software enables the researcher to build the desired model, including the modules of choice for G_1_, S and G_2_M in gen0, gen1 etc. (see supplementary Dataset S2).

The rendering of the proliferation works on cohorts of cells whose positions within G_1_, S or G_2_M are tracked by their age (time from entering) in that phase. Cells are grouped in each phase within age compartments of length Δ and straightforward balance equations connect the age distributions of cells at time t to those at time t+Δ (Figure S4). When a group of cells ends the cycle and divides, their offspring enter the next generation at the beginning of G_1_. This modelling of the cell flow through the cycle was extensively tested in previous studies [24]–[27] and is consistent with the general mathematical theory of age-structured cell populations [24]–[30], with the simple assumption of the balance of the number of cells entering, lost and exiting in each compartment. Recently, we introduced a separate simulation for cell populations of different generations, using this model to render the proliferation of a cell population by fitting at once FC and TL data [23]. The section “Modelling proliferation of untreated cells” recapitulates the model, including a further generalization for future uses. Using this model, cycling of the untreated IGROV-1 cell population was simulated (Figure S5) on the basis of average and coefficient of variation of the time spent in each phase and refined including quiescence characteristics for the cell line under study, calculated in preliminary TL and FC experiments, during asynchronous exponential growth. In addition to measuring cell cycle percentages and the number of cells in each generation, these experiments included the measure of intermitotic times, a detailed time course after BrdU pulse labelling and evaluation of quiescent cells by continuous BrdU labelling.

In order to render proliferation in the presence of an anticancer treatment, the basic proliferation model was nested with a model of the response to treatment, considering that the response can be very different not only in each cell cycle phase but also in subsequent generations. For this purpose, cycling of treated samples was simulated by perturbing the basic flow of untreated cells with modules that can be applied to any phase. Module types for G_1_ phase are shown in Figure 3, panels B to D (see Computational Methods for details). Modules render complex biological phenomena, e.g. the operation of a checkpoint in a specific cell cycle phase, with the minimum choice of parameters that enables quantifying the main antiproliferative effects (block, block recovery or death) occurring in that phase. The cycling cells death module (Figure 3B) mimics an immediate cell loss, possibly occurring at high doses with no detectable previous cell cycle arrest. The mitosis/polyploidy module (Figure 3C) deviates polyploid cells to a specific pool. Four different types of modules render checkpoint activity with increasing levels of complexity (Figure 3D). At the lowest level (type I), the effect is simply a delay of the progression in the phase where it is located. Type II acts permanently arresting a fraction of the cells transiting to the next phase in a specific compartment of blocked cells, which then may die or not, according to a “death probability” parameter. Type II can be applied together with a type I module, modelling a situation where some cells are permanently arrested and eventually die, while the others are simply delayed. Type III aims at rendering the competition between repair and cell death in blocked cells, adding a new parameter describing recycling to the type II module. Type IV adds further complexity to type III, introducing time-dependence for the onset of one or more of the effects (block, recycling or death). Parameters of each module are probabilities (e.g. a “block probability”) or rates (e.g. a “death rate”) of a specific event in the phase and generation where the checkpoint is located.

For any set of values of the input parameters of all modules, the software (supplementary Text S4) gives the simulation of the entire time course of the progression of the cell population within the phases and in subsequent generations, of both BrdU^−^ and BrdU^+^ cycles, and plots the results. Outputs are simulated data equivalent to those obtainable with different experiments, like cell cycle flow cytometry, time lapse imaging, Coulter counter and potentially any proliferation-based test (see Computational Methods).

### Optimization

**Figure 4:**
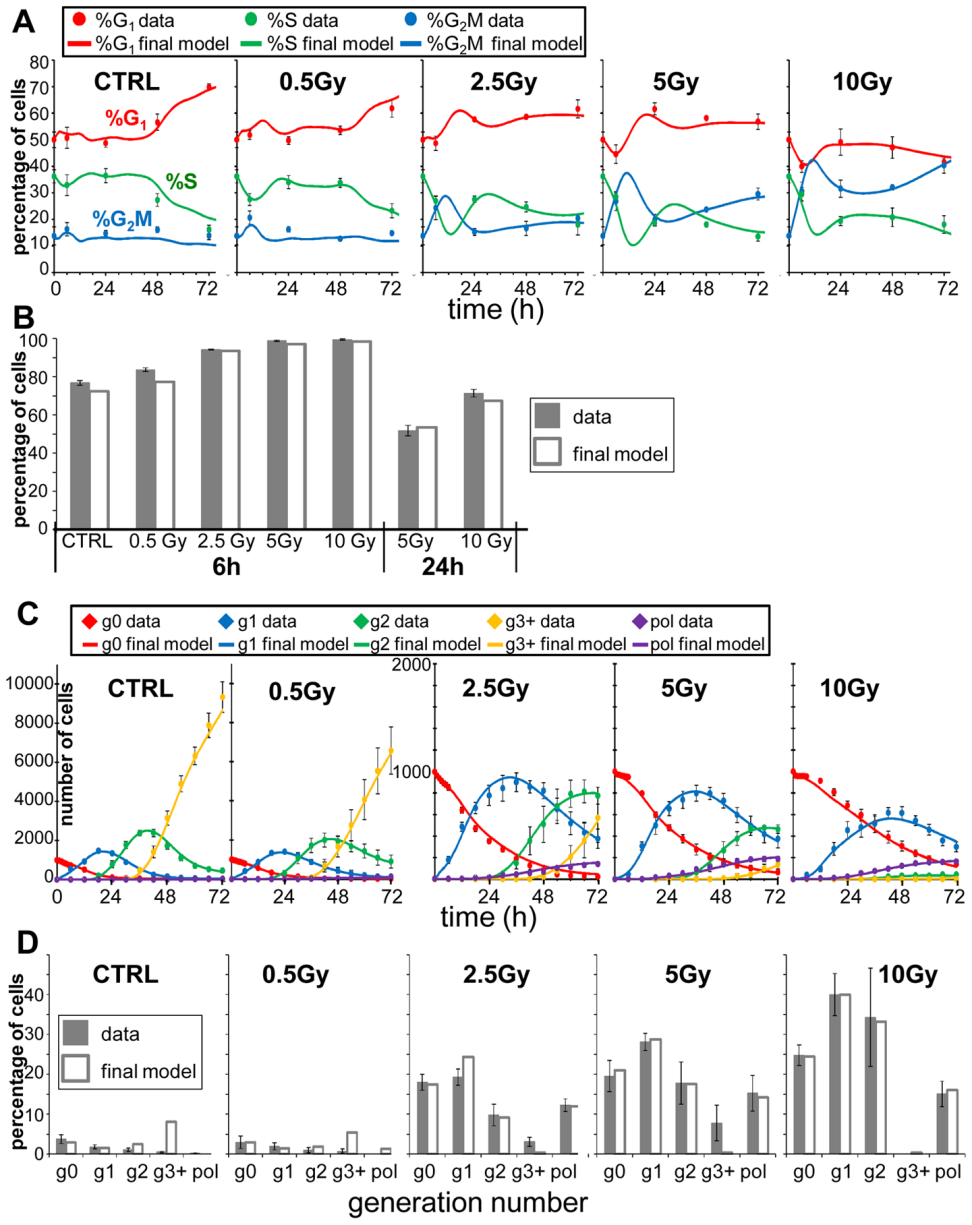
Data and fit with the final model. Time courses of measurable quantities obtained from the final model compared with experimental data (symbols), for each radiation dose. The good quality of the fit indicates that the model successfully predicts FC and TL data of all doses at the same time. a) Time course of %G_1_, %S, %G_2_M; b) percentage of residual undivided BrdU^+^ cells (supplementary Text S1); c) number of cells in gen0 (g0), gen1 (g1), gen2 (g2), gen3 and higher generations (g3+) and polyploid (pol), normalized assuming N(0) = 1000; d) percentage of cells which died in the 0–72 h observation time among cells entered in each generation. The symbols and error bars represent the mean and standard deviation of experimental data of at least three independent experiments (FC) or five replicate culture wells (TL).

**Figure 5:**
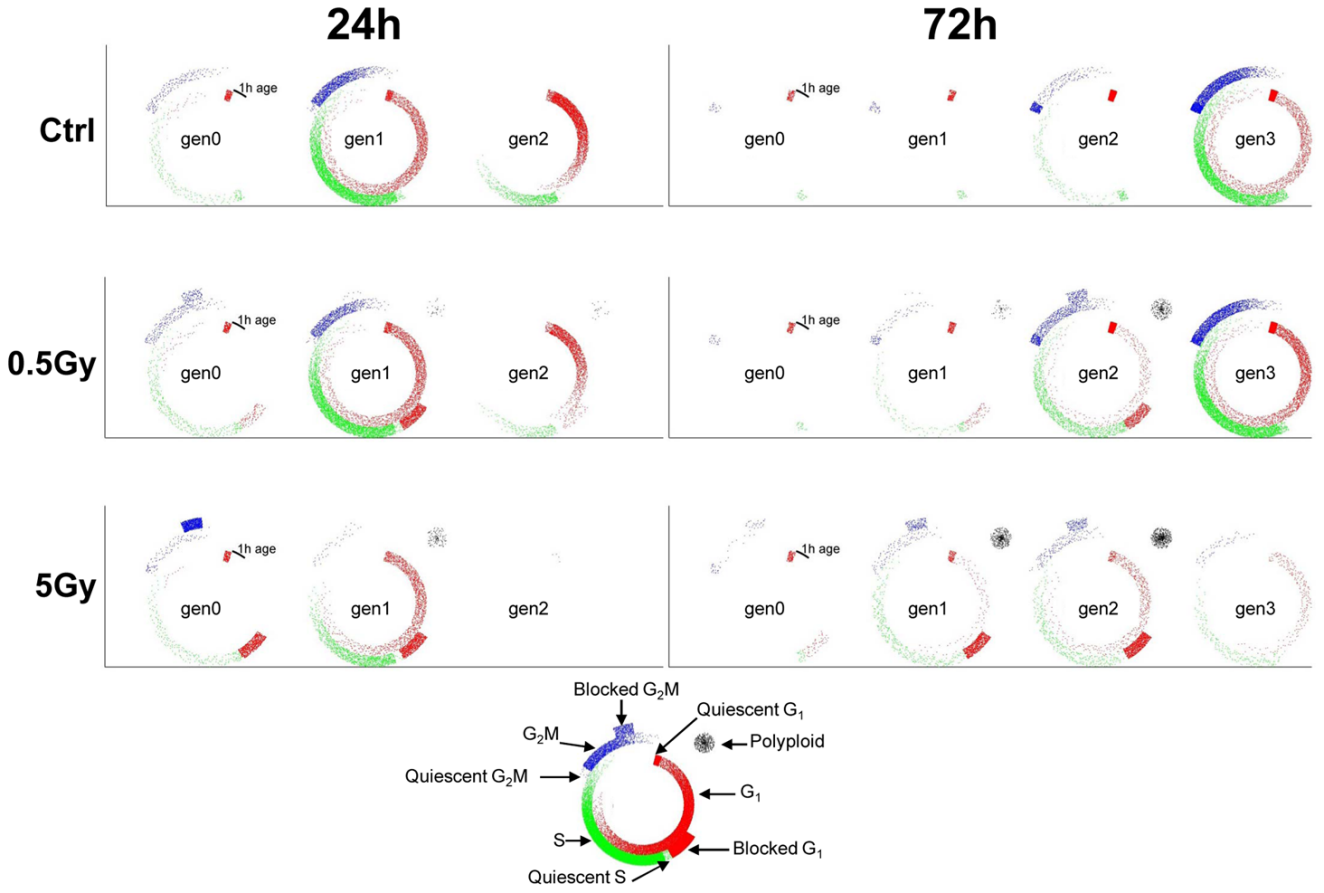
Dynamic rendering t of proliferation after X-ray exposure. Cell distributions in the cell cycle and over generations are shown at representative times (24 h and 72 h) and doses (ctrl, 0.5 and 5 Gy). The whole time courses for all doses (the final best fit model) are reported as Supplementary Videos. The plots shows the density of cycling cells in G_1_ (red dots in the inner sector), S (green, intermediate sector) and G_2_M (blue, external sector) phases according to their age (progressing clockwise from the starting point of each phase). The whole cycle lasts 24 h. The S and G_2_M starting points are placed in the figures at points corresponding to the mean duration of the previous phase, and are preceded by a compartment collecting quiescent cells. G_1_ blocked cells are presented as red dots in the intermediate sector, before the starting point of S phase, G_2_M-blocked cells are blue dots at the end of the cycle, external to the G_2_M sector. Polyploid cells are presented as black dots in a sector placed to the upper right of the cycle.

A rational workflow (Figure S6 and supplementary Text S4) was devised to build progressively the most suitable model of X-ray treatment, starting from the simplest and adding complexity when required, reaching a balance between the desired detail of rendering the process and the available data, avoiding over-simplification on one hand and over-parameterization on the other. Testing of tentative models was based on non-linear fitting of individual doses first, then performing a multi-dose fitting considering all data together. For multi-dose fitting, single dose parameters were constrained to obey simple dose-response relationships, using Hill or gamma functions. The workflow was successfully applied to fit simultaneously all FC and TL data of all doses in the experiment of X-ray exposure of IGROV-1 cells (Figure 4). The resulting best fit with the final model gives a dynamic (0–72 h) rendering of the flow of cells through the phases of the cell cycle and through subsequent generations, for controls and each X-ray dose (Videos S1 to S5). Figure 5 shows the cell cycle distribution at representative times and doses taken from the Movies 1 (control), 2 (0.5 Gy) and 4 (5 Gy). In particular the 0.5 Gy panels show that cells arrested in gen1 at 24 h were no longer there at 72 h, and most cells (85%) were cycling in gen3 at 72 h. Instead, at 72 h, 5 Gy-treated cells are distributed in the G_1_ and G_2_M blocks in gen1 and gen2 (40%) and in the polyploidy compartments (17%), with few (10%) cycling cells in gen3. The details of the dynamics of the process can be appreciated in the respective Supplementary Videos. Supplementary Dataset S3 includes FC and TL data of all X-rays doses and the user interface to simulate the final model, together with the instructions to download and run the simulation program as a Matlab standalone executable file (PaoSim_MultiDose).

### G_1_ and G_2_M block/repair/death in irradiated cells

**Figure 6:**
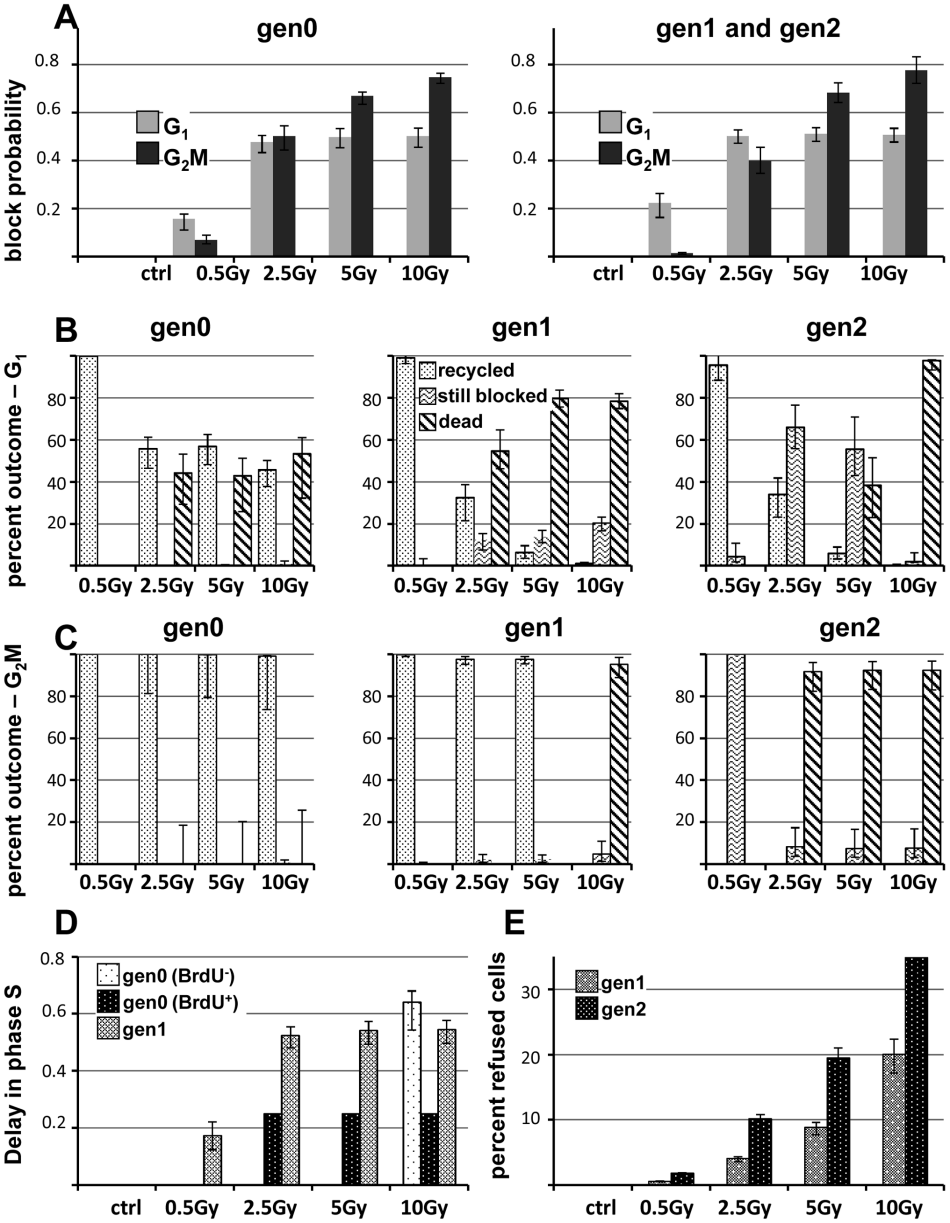
Checkpoint activities in the best-fit final model, as a function of the dose, in subsequent generations. (A), G_1_- and G_2_M-block probability (i.e. the fraction of cell intercepted and blocked at G_1_ and G_2_M checkpoints among cycling cells entering these phases) in irradiated cells (gen0) and their descendants (block probabilities in gen1 and gen2 were not distinguishable). (B) Outcome of G_1_-blocked cells, showing the percentages of cells that re-enter the cycle, die or remain blocked at 72 h in the indicated generations. (C) Outcome of G_2_M-blocked cells, symbols as in panels B. (D) Dose-dependence of the delay in phase S (fractional reduction of DNA synthesis rate) in gen0 and gen1. Data fit required a distinction between the delay of cells irradiated in S phase (BrdU^+^) and in G_1_/G_2_M (BrdU^−^) in gen0. No delay was found in gen2. (E) Polyploidization rate, as the percentage of cells that re-fused in gen1 and gen2. Error bars indicate 95% confidence intervals for parameter and derived quantities (e.g. fraction of blocked cells), calculated by fitting 1000 synthetic datasets generated by a Monte Carlo procedure (see Uncertainty Analysis in supplementary Text S4).

Rendering the proliferation *in silico* enabled us to retrieve information not available by direct observation and to quantify the processes in play. In particular, the best fit provided a quantitative evaluation of the activity of each checkpoint, separately in irradiated cells (gen0) and their descendants (Figure 6).

We found that the G_1_ checkpoint was activated in cells directly exposed to radiation at all doses (Figure 6A, gen0), inducing a partial G_1_ block immediately after exposure. Blocking activity was relatively weak at 0.5 Gy and high at 2.5 Gy, with no further increase at 5 and 10 Gy. G_2_M block probability was similar to that in G_1_ at 2.5 Gy, but was stronger and continued to increase at higher doses. Among cells reaching G_2_M in gen0 (i.e. not only cells originally in G_2_M but also those irradiated in G_1_ and S that progressed in the cycle) >95% were intercepted there at 10 Gy.

The fate of cells blocked in G_1_ and G_2_M was different. All G_1_-blocked cells were able to recycle only with 0.5 Gy, then gradually the recycling decreased and death increased, and at 10 Gy the majority of these cells eventually died (Figure 6B, gen0). In contrast, the balance between repair and death among G_2_M-blocked cells in gen0 was completely on the side of repair, as at least 95% of those cells eventually succeeded in dividing and entering gen1 also at the highest dose.

### G_1_ and G_2_M block/repair/death among descendants of irradiated cells

After the first division, cells irradiated with 0.5 Gy were minimally perturbed in gen1 and gen2, 25% of them being temporarily arrested or delayed in G_1_ (Figure 6A, gen1 and gen2). At higher doses, cell cycling was strongly hampered in all phases. 50% of the cells traversing G_1_ were intercepted and blocked there with 2.5 Gy or higher doses (Figure 6A, gen1 and gen2). Most of these G_1_-blocked cells died or were still arrested at 72 h (Figure 6B, gen1 and gen2). A dose-dependent G_2_M block was detected, reaching 0.8 probability at 10 Gy (Figure 6A, gen1 and gen2), with different outcome in gen1 and gen2. The majority of cells recovered from G_2_M block up to 5 Gy in gen1, while in gen2 most G_2_M-blocked cells died (Figure 6C, gen1 and gen2). At 10 Gy mortality in the G_2_M block was high in both generations.

### S checkpoint and polyploidization

The activity of S checkpoint is shown in Figure 6D, reporting the reduction of the rate of DNA synthesis. Gen0 cells exposed to X-rays while in S phase (BrdU^+^) were only moderately delayed in that phase and eventually reached G_2_M. The delay was stronger at 10 Gy for cells entering S after having overcome the G_1_ checkpoint (BrdU^−^), requiring more than 20 h on average to complete DNA synthesis. Cells in gen1 were then strongly delayed while traversing S phase at 2.5 Gy and higher doses, and no S checkpoint activity was observed in gen2.

Cell re-fusion was measured by the polyploidization module, acting on cells entering gen1 and gen2. Polyploidization was dose-dependent (Figure 6E), with about one third of re-fusions among cells that divided after 10 Gy. Most polyploid cells survived without dividing up to the end of observation, at 72 h, so that the fate of these cells remained unknown.

### *In silico* experiments

The final model can be used for *in silico* experiments, addressing specific questions.

**Figure 7:**
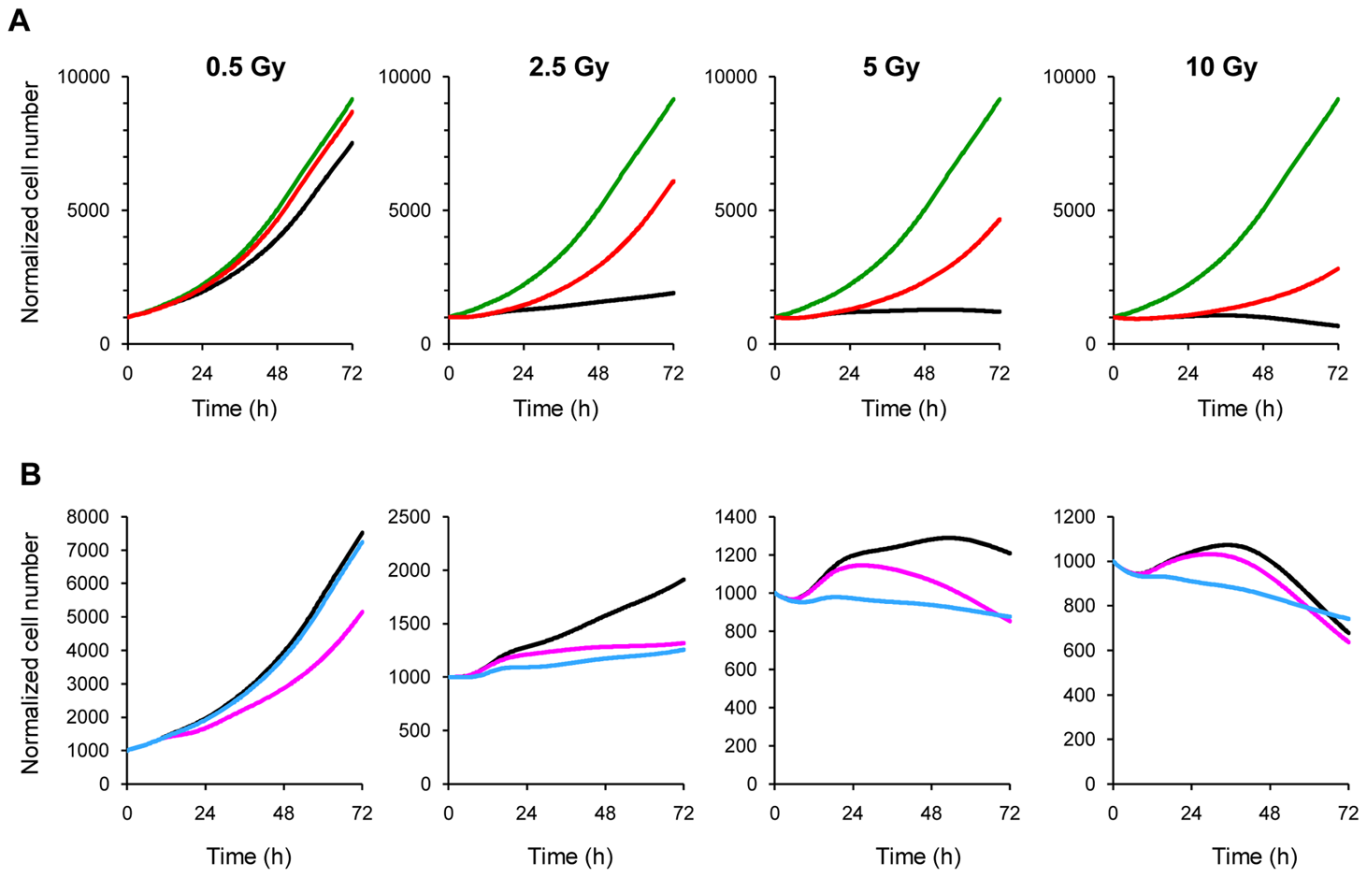
*In silico* experiments, exploring the consequences of default of specific checkpoints. (A) Simulated growth curve in the absence of perturbations in descendants (red line) compared to the best fit final model (black line) and proliferation in untreated cells (green line). (B) Growth curve in the final model (black line), in the presence of an additional agent (drug A) increasing the probability of G_1_ block (p = 0.8) (red line) and with an additional agent (drug B) preventing exit from G_2_M block (green line).

In a first experiment *in silico* we explored the impact on the overall proliferation of checkpoint activity in gen1 and gen2. We ran the simulation assuming the effects of radiation would be limited to the cells directly exposed to X-rays, with no effects on their descendants. In this scenario, we found that cancer cell expansion was not stopped even at the highest dose (Figure 7A), demonstrating that the events in gen1 and gen2 are crucial for the response to treatment.

At the lowest dose, in the absence of effects on descendants, the growth curve almost overlaps the growth of untreated cells, so most of the modest growth inhibition has to be attributed to perturbations in gen1 and gen2. As the dose increases, the perturbations in gen0 become more obvious, but cell cycle checkpoint activities on the descendants of irradiated cells still make a major contribution to the overall effect.

Other *in silico* experiments were run strengthening the G_1_ block or preventing the exit from G_2_M block, miming hypothetical co-treatments with agents eliciting these specific effects in combination with X-rays. Figure 7B shows the effects of co-treatments with two hypothetical drugs: drug A acting in G_1_, resulting in potentiation of the G_1_ block, drug B acting in G_2_M, inhibiting recycling from that phase, possibly acting on one of the stages of the repair process.

At 0.5 Gy the effect of drug B is irrelevant, in view of the limited importance of the G_2_M checkpoint at this dose, while drug A, acting on G_1_, would potentiate the X-ray effects. At 2.5 Gy drug B would potentiate X-rays more than drug A in the short term, but in a long term the differences tend to disappear and eventually re-growth would be similar in both instances. At very high doses, the effects of drug A would become irrelevant, because the G_1_ checkpoint is already strong with X-rays alone. Drug B would be more effective in the short term, but less on a longer term, as it would prevent cells reaching the G_1_ checkpoint in gen1 and gen2 where cytolethal activity was greater.

The results suggest that strengthening the G_1_ block would potentiate the effects of radiation, but only at intermediate doses. A drug preventing the exit from G_2_M block would be more effective in the short term, but less on a longer term.

## Discussion

Mathematical modelling of biological or biomedical systems is now facing the challenge of quantitative rendering of the biological phenomena, taking into consideration the vast amounts of data produced by experimental biomedicine, with appreciation of the biological complexity, with its dynamics, and the processes of measure, with their strengths and limitations.

Also in cancer research the demand for proper quantitative interpretation of the processes in play is rising [31], [32]. The method we propose enables a detailed study of the mechanisms concurring to build the response to anticancer treatment at the “cell population” level, where measures were made on single cells and on a statistically representative number of them, accounting for inter-cell variability. This provides a different view respect to that obtained with bulk measures on cell homogenates, representing “average” quantities, unpredictably weighted by the relative numerosity of cell subsets with different amounts of the measured quantity. At the cell population level, we may exploit a very rich amount of information, coming from established or new techniques. It remains a challenge to find the way to organize all that we can measure, and possibly put together complementary information from different techniques, in order to give sense to the observed heterogeneity. This challenge must be addressed, if we really aim to reach an unbiased picture of the behaviour of a multicellular system, from *in vitro* cultures to *in vivo* organs or tumours.

We demonstrated that it is possible to integrate FC and TL platforms via *in silico* rendering of the dynamics of the underlying cell cycle progression. This was achieved with a flexible model-building environment, which enabled us to challenge a variety of tentative models in a manageable time. Once the model of unperturbed proliferation was established, we progressed to study how the system responds to a hypothetical treatment. Different types of modules were implemented in the model, miming the activity of cell cycle controls at increasing levels of complexity. The user can select a specific effect and look at its consequences in simulated experiments.

Wishing to build a model for a real treatment, we strengthened the parameter estimation by fitting data from different treatment levels (four X-ray doses in our example) simultaneously and optimizing the dose-response for each parameter, with simple regularity assumptions. This combination of data from different doses and platforms provided internal cross-validation to exclude the existence of multiple solutions in models including several variable parameters. Our optimization procedure initially establishes a relatively simple model with a unique best fit solution, which was then cautiously refined, up to the definition of a final model, by which the data of all doses were fitted with average errors 1.6% (FC cell cycle percentages) and 3.6% (TL cell generation numbers) (Figure 4). The final model provided a unifying view of the effects of radiation that puts in order and defines the relative importance at the different doses of the main phenomena in play, which are described separately in the literature [21], [22], [33], [34] (supplementary Text S5).

Study of the final model disclosed the dynamics of checkpoint activities and their dose-dependence, showing up basic differences between G_1_ and G_2_M checkpoint responses that were not perceivable by simple data inspection. In particular, G_2_M-blocked cells were eventually able to recover from the block and divide even after treatment with 10 Gy, while at this dose the majority of G_1_-blocked cells eventually died there. The response was not restricted to gen0 cells, but cell cycle progression was also hampered in gen1 and gen2. Our results strongly support the concept of a limited efficiency of a single checkpoint to process the damage [35], suggesting that cycling of individual cells may be subsequently hampered in different phases. The model was also used as a basis to explore, with in silico experiments, the consequences of default or potentiation of specific checkpoints, which in principle could become feasible with new, molecularly targeted co-treatments, or may occur in tumours with particular genetic defects. Obviously, the real response to treatment is even more complex than the proposed model. It does not include, for instance, possible correlations between sibling cells for probabilities of specific outcomes, or signalling between cells or spatial issues driving neighbour cells towards a common fate. Still our model works at a quite high complexity level, including details of the effects in each phase and generation at different treatment doses. This was made possible avoiding over-parametrization only through fitting of a very rich and complex data set. Further details would require more parameters and additional data, which are not precluded in future studies.

## Materials and Methods

### Experimental methods

#### Cell culture and treatment

Cells from a human ovarian carcinoma line (IGROV-1) were grown in RPMI-1640 medium (Biowest) as described before [36]. For these experiments, IGROV-1 cells were seeded in six-well plates (Iwaki) and after two days, while in exponential growth, they were irradiated with a RadGil (Gilardoni) 200 kVp X-ray machine at a dose rate of 1.2 Gy/min. The experiments were conducted in parallel by time-lapse microscopy and flow cytometry. The same experimental protocol was adopted for controls and 0.5, 2.5, 5 or 10 Gy exposures, spanning the range from low to high efficacy based on preliminary tests.

#### Flow cytometry

For flow cytometry, cells from replicated samples were collected at the specified times, pooled, fixed in cold 70% ethanol and stained. We applied two flow cytometric techniques: (i) monoparametric cell cycle analysis and (ii) 5′-bromo-2′-deoxyuridine (BrdU) pulse labeling. For analysis of DNA content a suspension of permeabilized cells was stained with propidium iodide (PI) (Calbiochem) [36]. Short-term perturbations were investigated by BrdU pulse-chase analysis. Pulse labelling was obtained adding 30 µM BrdU (Sigma) to the cell cultures for 15 minutes. After thorough BrdU washout, the cells were irradiated and incubated in BrdU-free medium for the indicated times, then harvested, fixed and stained with PI and BrdU monoclonal antibody (Becton Dickinson) [36]. Incorporation of BrdU instead of thymidine during DNA replication enabled to analyse separately cells in S-phase (BrdU^+^), from those in G_1_/G_2_M (BrdU^−^) at the time of the pulse.

#### Time-lapse microscopy

By time-lapse microscopy we snapped the same field of view at discrete time intervals. Cells were seeded in six-well plates (Iwaki), irradiated while in exponential growth, then placed on a time-lapse instrument to capture transmission-phase images from multi-well plates. The Imaging Station cell∧R (Olympus) used for these time-lapse experiments consists of: a) X81 motorized inverted microscope (Olympus) fitted with an incubator to maintain 37°C, 5% CO_2_ and 60% humidity (OKOlab); b) ORCA-ER CCD camera (Hamamatsu); c) X-Y positioning stage. All images were collected with an UPlanFLN 10× (Ph1) objective with 0.30 NA. Sequences were captured every 20 minutes for 72 h, and for the same field in each well.

Time-lapse microscopy movies were analysed using free ImageJ software (W. Rasband, National Institute of Health), tracking all cells in the fields of view (50–100 cells per field) and their descendants, using a modified version of the “Manual Tracking” plug-in distributed with ImageJ.

### Modelling proliferation of untreated cells

Asynchronous proliferation of untreated cell populations in the biological system is achieved by a balance of the cell cycling process, quiescence and cell death [37]. The multi-generation cell cycle model, with variable phase durations but without quiescence and death, is shown in Figure S4 and Supplementary Text S3. State variables are *G_1_*(k,t,gen_i_), *S*(k,t,gen_i_), *G_2_M*(k,t,gen_i_), giving the number of cells in generation gen_i_, with age k at time t in the respective cell cycle phases. Model parameters are (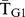, CV_G1_, 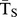, CV_S_, 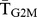, CV_G2M_), i.e. the average and coefficient of variation of the phase durations, on which basis the exit probability for each compartment (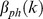) were calculated (see supplementary Text S3). Because quiescence and spontaneous cell death are often not negligible even in untreated cell populations growing *in vitro* in optimal environmental situations, quiescence modules were included in G_1_, S and G_2_M to render these phenomena.

#### Quiescence module

Parameters: Quiescence probability (*pQ_ph_*), Death rate (*DRQ_ph_*).

Quiescent cells are localized in a distinct Q compartment in each phase, and their numbers *G_1_Q*(t), *SQ*(t) and *G_2_MQ*(t) add to the list of the state variables of the basic model shown in Figure S4. The probability that a cell entering G_1_, S or G_2_M phase becomes quiescent is represented by pQ_G1_, pQ_S_ and pQ_G2M_ respectively. Cell death among quiescent cells due to spontaneous processes is included in the model by parameters DRQ_G1_, DRQ_S_, DRQ_G2M_.

The module can be used to render the approach to confluence, a phenomenon that should be taken into account when the observation lasts several days. During this process, the fraction of quiescent cells and death events increase, progressively shifting the distribution equilibrium, reducing the fraction of cycling cells. This was modelled by increasing pQ, (specifically pQ_G1_ as %G_1_ increases approaching confluence) using a Hill function linked to the overall cell number. The multi-generation model was adopted in recent publications to fit both FC and TL data in untreated cell cultures, including refinements describing the approach to confluence [38]. More details of the procedure of fitting of untreated cells are reported there.

#### Further generalization: The multi-cycle model

We currently use as basis of treatment modelling a multi-cycle model which is a generalisation of the multi-generation model of untreated cell populations. In the multi-cycle model the connection between subsequent cycles is not fixed, with a fraction of newborns (parameter *pOut_i_*) entering cycle “i+1” while the others (i.e. a fraction 1-*pOut_i_*) re-enter in the same cycle “i” of their mother cells (“i” ranges from 0, representing the first cycle, to “f”, representing the final or last cycle considered). This provides flexibility to the user, who can be interested to model different situations:

- by setting *pOut_0_* = 0 cells continue to re-cycle in cycle 0, obtaining a single-cycle model (cells after division re-enter in the same cycle and generations were mixed);
- by setting *pOut_i_* = 1, for i = 0 to f−1, cells enter subsequent cycles, intended as “generations”, obtaining the multi-generation model. Then, for the last generation “f” *pOut_f_* = 0 would make the cells of “f” and subsequent generations to be pooled together in the final cycle. Instead setting *pOut_f_* = 1, cells of generation f+1 would not be considered;
- by setting *pOut* parameters to any value it becomes possible to model differentiation chains, intending the cycles as “differentitiation stages” and *pOut_i_* as the fraction of dividing stage “i” cells that differentiate to the (more mature) stage “i+1”. Although these situations may require additional features (e.g. feedback controls between stages) the multi-cycle model could be a convenient framework, in which such features could be implemented in the future.

The dynamics of the multi-cycle model is provided by the following balance equations (the suffix “gen” is replaced by “cyc”, cyc_i_ referring to the cycle “i”) :
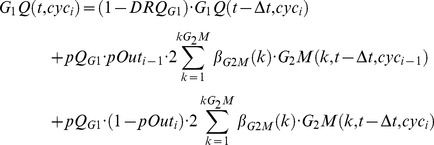
(1)

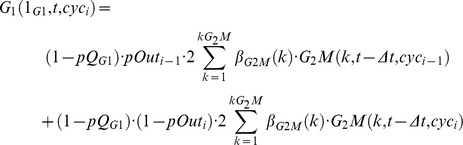


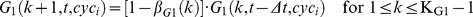


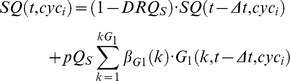


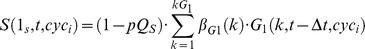


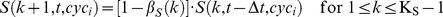


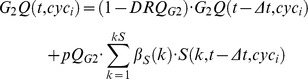


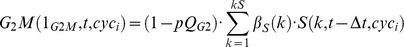


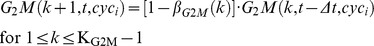
where the terms containing cyc_i−1_ should be omitted in the cyc_0_ equations.

An additional feature already implemented in the model is the G0 sub-phase, that is a single-compartment phase set before G_1_ of each cycle. Differently than definitively quiescent cells, modelled with the above quiescence module, G0 cells may leave G0, with first order output kinetics, and enter G_1_. This enables the user to include classical probability transition cell cycle models [39] within our framework. The parameters associated to the G0 phase are: γ (rate of exit from G0 to G_1_); θ (probability to by-pass G0 to enter directly in G_1_) [40] and DR_G0_ (death rate of G0 cells). The balance equation for G0 adds to eqs. 1:
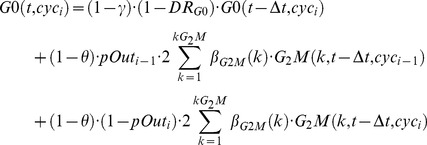
(1bis)With straightforwards corresponding changes in the entry in G_1_ and G_1_Q.

In this way, the user is free to build and compare various cell cycle models, including G0 and G_1_, S and G_2_M phases with not fixed duration.

### Starting age distribution of cells

Simulation is intended to start at a laboratory time (0 h) corresponding to the start of a treatment in the experimental setting. Thus the model requires the input of the age distributions at 0 h (G_1_(k,0,0), S(k,0,0) and G_2_M(k,0,0)) from which the time evolution is simulated according to the previous equations. The user can provide any starting distribution, but we chose the asynchronous distribution in all our studies aiming at rendering experiments with non-synchronised cell populations. Any cell population, under constant environmental conditions, reaches asynchronous exponential growth, and good laboratory practice requires that cells are in this condition (or as near as possible) before an *in vitro* treatment, for data reproducibility. In this condition the probability distribution of cells in the cell cycle phases and ages is time-independent. A desynchronization routine of our program automatically calculates the asynchronous distribution, by running the cell cycling procedure from an approximated initial distribution until the cell cycle percentages varied by less than the desired precision (0.1% in the present study). Then the G_1_, S, and G_2_M age distributions were normalized in order to start the cell cycle simulation with a given number of cells at 0 h (e.g. 1000 cells), providing G_1_(k,0,0), S(k,0,0) and G_2_(k,0,0). A program performing the desynchronization routine is freely available as specified in supplementary Dataset S1 (see also the Software section in Supplementary Text S4).

To simulate pulse-chase BrdU experiments, we run two cell cycle simulations independently for BrdU^−^ cells and BrdU^+^ cells. The effect of pulse labeling was reproduced by splitting the starting distribution, assigning G_1_(k,0,0) and G_2_(k,0,0) (plus quiescent cells) to the BrdU^−^ cycle and S(k,0,0) to the BrdU^+^ cycle. Thereafter the two cell cycle simulations proceeded independently. The program can also reproduce the effect of labeling of any duration (if required for specific experiments, e.g. with continuous BrdU labeling), transferring cells entering S phase from the BrdU^−^ to the BrdU^+^ cycle up to the time when BrdU is removed. These features guarantee maximum flexibility to the program, to simulate different experimental plans.

### Connecting the model to experimental data

At the end of a simulation run, the program derives the time courses of several kinds of simulated data with simple calculations from the time courses of the state variables. First, the proportions of cells in G_1_, S and G_2_M are trivially calculated by summing the number of cells in all compartments of that phase and dividing by the total number of cells. These predictions are compared with experimental FC percentages at the measurement times. Then the program calculates the time courses of the number of cells in each generation (N_gen(i)_(t)), summing up the number of cells in all compartments of all phases of the generation. Predicted N_gen(i)_(t)s are directly compared with the corresponding TL data.

The time course of overall cell number (N(t)) is also calculated, summing the number of cells in all generations, and can be compared with experimental measures obtained by TL or other techniques (e.g. Coulter counter). Similarly, simulated percentages of cells in the BrdU^−^ and BrdU^+^ subsets are calculated summing the number of cells in the appropriate subset compartments and plotted with experimental counterparts. The time course of the percentages of undivided BrdU^+^ cells (%Und^+^(t)) is obtained by summing the numbers of cells in all gen0 compartments of the BrdU^+^ cell cycle and dividing by the overall cell number.

Another valuable item is the the distribution of Tc, measurable by TL. It comes from frequency measures of intermitotic times in a sampling of the whole population (we had a total of 2480 cells), which were normalized and assumed to be representative of the distribution of Tc in the whole population (experimental F(Tc)). While the simulated average 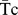 is easily calculated directly in our model as 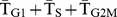, and compared with the corresponding datum, a specific routine was designed to calculate the whole F(Tc), on the basis of *F_G1_(k)*, *F_S_(k)* and *F_G2M_(k)*. Considering two phases, e.g. S and G_2_M, the joint probability that a cell traverses S and G_2_M with times *k_S_* and *k_G2M_* is *F_S_(k_S_)*×*F_G2M_(k_G2M_)*. Thus the probability that the duration of S+G_2_M is *k_SG2M_* is given by the formula: *F(k_SG2M_)* = ∑*F_S_(k_S_)*×*F_G2M_(k_G2M_)* where the summation includes all combinations of *k_S_* and *k_G2M_*, such that *k_S_*+*k_G2M_* = *k_SG2M_*. Then F(Tc) is calculated as *F(k_G1SG2M_)* = ∑ *F_G1_(k_G1_)*×*F(k_SG2M_)* where the summation includes all combinations of *k_G1_* and *k_SG2M_*, such that *k_G1_*+*k_SG2M_* = *k_G1SG2M_*.

Simulated data are derived in the same way when perturbation modules are included except in the case of F(Tc), which was calculated only for untreated cells.

Summarizing the simulation procedure, upon input of the parameter values, the program: i) calculates *F_ph_(k)* and *β_ph_(k)*, ii) runs the subroutine to produce the asynchronous starting cell distribution, iii) simulates the movement of the cells through the cell cycle in subsequent generations, calculating the time course of the state variables up to the desired end-time, and iv) calculates the time course of all derived quantities (such as %G_1_(t), %S(t) and %G_2_M(t), %BrdU^+^(t), %Und+(t), 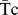, F(Tc), N_gen(i)_(t) etc.) and updates their plots with the corresponding experimental data.

### Modelling proliferation of treated cells

Perturbations of cell cycling induced by treatment are modelled with modules for G_1_, S and G_2_M response in the gen0, gen1, gen2 etc., superimposing and modifying the “unperturbed” flow of the cell through the cycle. An additional module governs polyploidization at the end of each generation.

The user can choose among several modules types, emulating different biological effects, and locate them in any phase and generation, so that it is possible to deal with the different antiproliferative effects of anticancer treatments without modifications of the software. Supplementary Dataset S2 includes the user interface to build a single-dose model, together with the instructions to download and run the simulation program as a Matlab standalone executable file (PaoSim_SingleDose). The interested reader can also test the model for untreated cells using the PaoSim_SingleDose program with no perturbation modules.

During the optimization procedure, the choice of the module and module type for each phase was guided by the law of parsimony: in general we applied the module type that satisfactorily reproduced the data with the smallest number of parameters. The optimization procedure therefore involved several steps, as described in supplementary Text S4.

#### Checkpoint modules

We implemented four types of checkpoint modules, aiming to render the activity of a checkpoint with increasing levels of complexity:

- Checkpoint Module Type I. Parameter: Delay (*Del_ph_*).
- Checkpoint Module Type II. Parameters: Block probability (*pBL_ph_*), Death rate (*DRBL_ph_*).
- Checkpoint Module Type III. Parameters: Block probability (*pBL_ph_*), Death rate (*DRBL_ph_*), Recycling rate (*Rec_ph_*).
- Checkpoint Module Type IV. Parameters: pBL_ph_(t) (pBL_ph_max, pBL_ph_min), DRBL_ph_(t) (DRBL_ph_max,DRBL_ph_min), Rec_ph_(t) (Rec_ph_max, Rec_ph_min).

With checkpoint module type I, cell cycle progression is hampered in the phase where the module is located, resulting in a longer phase duration. Cell death is not considered and each type I module has only one input parameter, rendering a progression delay within a phase. *Del_ph_* is comprised between 0 (no effect) and 1, the latter indicating complete cell “freezing” within that phase, and affects the average duration of a phase according to the formula:
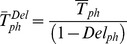
where 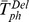 is the average duration of a phase “*ph*” (either G_1_, S or G_2_M) in the presence of delay and 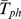 in its absence. The delay parameter in phase S is equivalent to the fractional reduction of average DNA synthesis rate in replication, so equation (2) can be rewritten as
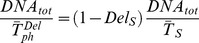
where DNA_tot_ is the total amount of DNA to be replicated. Similarly, *Del_G1_* and *Del_G2M_* emulate a lengthening of the respective phases with a generic connection with the underlying molecular events tracking the maturation within G_1_ or G_2_M.

In the model, this checkpoint module acts on all age compartments of a phase, keeping in the original compartments a fraction (*Del_ph_*) of the cells that should have moved to the next (i.e. from k to k+1) in a given step-time. Thus, the *ph(k,t, gen_i_)* equations (1) of the number of cells in compartment k of the generic phase “*ph*” are modified by this module as follows:
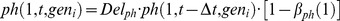


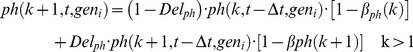
(3)It is possible to demonstrate by simulation that this modelling prolongs the phase duration specified by equation (2), dealing also with the limit case *Del_ph_* = 1.

If only modules of this kind are applied in a given generation, the average cell cycle can be calculated with the formula:
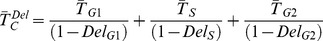
(4)

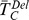
 compares with the corresponding experimental datum obtained in TL.

Checkpoint module type II emulates cell killing induced by treatment assuming that part of the cells passing through a phase are first arrested then possibly die. *pBL_ph_* represents the probability that a cell does not exit a phase, becoming definitively blocked there. The simulation diverts a corresponding fraction of cells, among those completing a phase at a given time, into a separate compartment of blocked cells. *DRBL_ph_* is the probability of dying, among blocked cells, in a step-time. The model makes no distinction between different death processes (apoptosis, necrosis, etc.), and only considers the final loss of the dead cells from the number of counted cells. Additional balance equations for the number of blocked cells (B_ph_(t, gen_i_)) were included as follows:
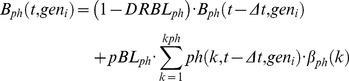
(5)with consequent modifications of the equations for quiescence and the first compartment of the next phase, to take account of the reduced entry due to the cells blocked in the previous phase.

Checkpoint module type II is compatible and can be joined with type I, to describe the effect of treatment with three parameters (*Del_ph_*, *pBL_ph_*, *DRBL_ph_*).

Type III modules include the connections between block, recycling and killing, assuming three alternative fates for cells intercepted at the checkpoint: i) to repair the damage and recycle, entering the next phase, ii) to die at the checkpoint, or iii) to remain definitively blocked. Rec_ph_ is the probability that a blocked cell re-enters the cell cycle in a step-time. This parameter is indicative of possible repair that can occur in cells blocked at each checkpoint. *pBL_ph_* is the probability of being intercepted by the checkpoint for cells passing through a phase, including cells definitively or temporarily blocked in that phase; as such it is a measure of the strength of the checkpoint itself (differently from the block parameter in model II). In the *in silico* rendering of the process, a fraction of cells corresponding to the Block probability is deviated into the block compartment, where they may die, according to the Death rate, or exit, according to the Recycling rate. The corresponding equation for the compartment of blocked cells was modified as follows:
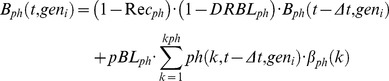
(6)and the pool of cells entering the next phase was accordingly modified, to take into account blocked and recycled cells.

With type III the increase of Tc is the consequence of the dynamics of entering/exiting the block, emulating the biological process of G_1_ and G_2_M checkpoints in a more realistic way than the Delay parameter used in type I modules, which are better to render hampering DNA replication caused by the S checkpoint. However, differently from type I, no simple relationship can be drawn between the values of a type III and 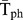.

Type IV is a refinement of type III. We considered time-dependent parameters to render a delay between block and subsequent recycling or death rate. We modelled the time dependence with a Hill function, as
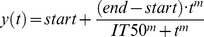
where t is time, y is either pBL_ph_(t), DRBL_ph_(t) or Rec_ph_(t), *start* and *end* are initial (t = 0) and final (asymptotic) values, IT50 the time when half the maximum is reached and the sigmoidicity “m” measures the steepness of the curve. In the present studies we kept the parameters IT50 and m fixed, with values based on inspection of the data and on simulation tests. In addition, to avoid over-parameterization, we applied this module only in gen0, when the data provided compelling evidence for time-dependence of recycling.

#### Cycling-cells death module

Parameters: Cycling cells death rate (*DR_ph_*).

An additional module was required to model immediate cell death, possibly occurring at high doses with no detectable previous cell cycle arrest. This was trivially obtained with a single death rate parameter (DR_ph_), acting on all non-blocked cells in a phase. Because this module was designed to model immediate cell death, it was used only in gen0.

#### Mitosis module

Parameter: m = 2 (fixed).

The mitotic module in the present study simply doubles the number of cells that ends a cycle before they enter the next generation. In general, m<2 would render cell death in mitosis.

#### Polyploid module

Parameters: Polyploidization probability (*pPol*), Polyploid cells death rate (*DRPol*).

Polyploidization was modelled introducing the parameter *pPol*, representing the probability that newborn cells re-fuse together, and the corresponding death rate (DRPol). The polyploid module assumes that a fraction (pPol) of newborns re-fuse, collecting re-fused (polyploid) cells in a separate compartment, where they die at a rate DRPol. Polyploid cells were localized in a new compartment and the equation for the number of polyploid cells (*Pol(t, gen_i_)*, for gen1 and higher generations) was:
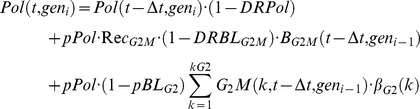
(7)with consequent changes of equations for *G_1_Q(t, gen_i_)* and *G_1_(1_G1_, t, gen_i_)*.

A refinement of this module led us to introduce a time-dependence of *pPol*, with a Hill function as in checkpoint module type IV.

## Supporting Information

Dataset S1 **User interface for the desynchronization routine, including instructions to download and run the Matlab standalone executable file (PaoSim_Desync) that performs the simulation.**
(XLS)

Dataset S2 **User interface for building a single-dose model, including instructions to download and run the Matlab standalone executable file (PaoSim_SingleDose) that performs the simulation.**
(XLS)

Dataset S3 **User interface for the final multi-dose model, including instructions to download and run the Matlab standalone executable file (PaoSim_MultiDose) that performs the simulation.** This dataset includes FC and TL data of IGROV-1 cells treated with X-rays as described in the main text.
(XLS)

Figure S1 **Main statistics of TL experiments.** The cumulative distribution of the time to division in gen0 (panel A) and the average Tc in gen1 and gen2 (panel B) were indicative of cell cycle delays, the percentage of dead cells in each generation in the whole 0–72 h observation time (panel C) of the cytolethal effect, the percentage of re-fused cells (panel D) of the polyploidization. Columns and error bars in panels B, C and D represent mean and standard deviation respectively, in at least five independent culture wells. All wells were pooled in panel A.
(TIF)

Figure S2 **Main results of FC experiments: DNA histograms.** Abscissa is proportional to cellular DNA content, with G_1_ and G_2_M cells in the positions indicated. Signals below the G_1_ peak indicate the presence of cell debris, at doses and times consistent with cell death observed with TL. Signals above the G_2_M peak indicate tetraploid cells, again confirming TL observations.
(TIF)

Figure S3 **Main results of pulse-chase BrdU experiments.** Representative dot plots for a pulse-chase BrdU experiment, taken at 6 h (upper panels) or 24 h (lower panels). Abscissa: cellular DNA content measured by PI fluorescence. The positions of G_1_ and G_2_M are indicated. Ordinate: cellular BrdU content measured by Anti-BrdU and a secondary FITC-labeled antibody. The lines mark the region of interest, separating BrdU^+^ from BrdU^−^ and divided from undivided BrdU^+^ cell subpopulations.
(TIF)

Figure S4
**Basic cell cycle model with variable phase durations.** Cells enter the first age compartment (0–0.5 h) in a phase “ph” (G_1_, S or G_2_M) then gradually progress through the subsequent age compartments, while other cohorts enter the phase. Because the time spent in a phase (T_ph_) is variable for the cells of the cohort, when the cohort reaches a given age, it has been depleted of the cells that have already completed the phase and a further fraction (β_ph_) of the remaining is expected to exit the phase at that age. The exit probability β_ph_ is a function of age that univocally depends on the average (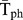) and coefficient of variation (*CV_ph_*) of the phase durations. At a given time, some cells from all age cohorts complete the phase, collectively forming the pool of exiting cells that will enter the next phase model at the next time.(TIF)

Figure S5 **Model of IGROV-1 proliferation (asynchronous growth).** Data from preliminary experiments with untreated IGROV-1 cells during exponential growth were fitted with the model described in Computational Methods. Further refinements were included when fitting departed from exponential growth (e.g. the approach to confluence) as reported elsewhere [38] [23]. Left panels: best fit parameters. Main parameters were the average phase durations and their CV (with the frequency distributions of T_G1_, T_S_ and T_G2M_ shown in the lower panel). Additional parameters (probabilities of quiescence: pQ_G1_, pQ_S_, pQ_G2M_ and death rate: DRQ) were included to explain the small percentage (4–8%) of quiescent cells observed by BrdU continuous labelling or TL and spontaneous death (1–3% per generation) observed by TL, as required to fit simultaneously all data shown in the right panels. Right panels: experimental data (symbols) and simulation (continuous lines) including: A) cell cycle percentages from monoparametric FC, B) BrdU^+^, Und+ and %Res^+^ (percentage undivided among initially labelled cells, see supplementary Text S1) from FC BrdU pulse chase experiments, C) time course of the number of cells in each generation (normalized assuming N(0) = 1000) from TL, D) frequency distribution of intermitotic times from TL (2480 cells).
(TIF)

Figure S6 **Flow chart of the optimization procedure.**
(TIF)

Table S1 **Qualitative inspection of TL data.**
(DOC)

Table S2 **Modules and parameters of the single-dose model A.**
(DOC)

Table S3 **Dose-dependence and variable coefficients in multi-dose model A.**
(DOC)

Table S4 **Dose-dependence and variable coefficients in the final model.**
(DOC)

Table S5 **Best fit values of variable parameters in the final model, with 95% confidence intervals.**
(DOC)

Text S1 **First-level data analyses.**
(DOC)

Text S2 **Data inspection.**
(DOC)

Text S3 **Modelling proliferation of untreated cells.**
(DOC)

Text S4 **Optimization procedure and softwares.**
(DOC)

Text S5 **Antiproliferative response to X-ray exposure.**
(DOC)

Video S1 **Movie of a representative control sample obtained by TL microscopy.** Only one frame per hour is shown to reduce file size.
(AVI)

Video S2 **Movie of a representative treated sample (5 Gy) obtained by TL microscopy.** Only one frame per hour is shown to reduce file size.
(AVI)

Video S3 **Computer rendering of cell cycle progression in untreated cells.**
(AVI)

Video S4 **Computer rendering of cell cycle progression after 0.5 Gy exposure.**
(AVI)

Video S5 **Computer rendering of cell cycle progression after 2.5 Gy exposure.**
(AVI)

Video S6 **Computer rendering of cell cycle progression after 5 Gy exposure.**
(AVI)

Video S7 **Computer rendering of cell cycle progression after 10 Gy exposure.**
(AVI)
