# Supplementary figures and images for: Dynamic Rendering of the Heterogeneous Cell Response to Anticancer Treatments
Source: PLoS Comput Biol. 2013 Oct 17;9(10):e1003293. doi: 10.1371/journal.pcbi.1003293 (PMC3798276; doi:10.1371/journal.pcbi.1003293)

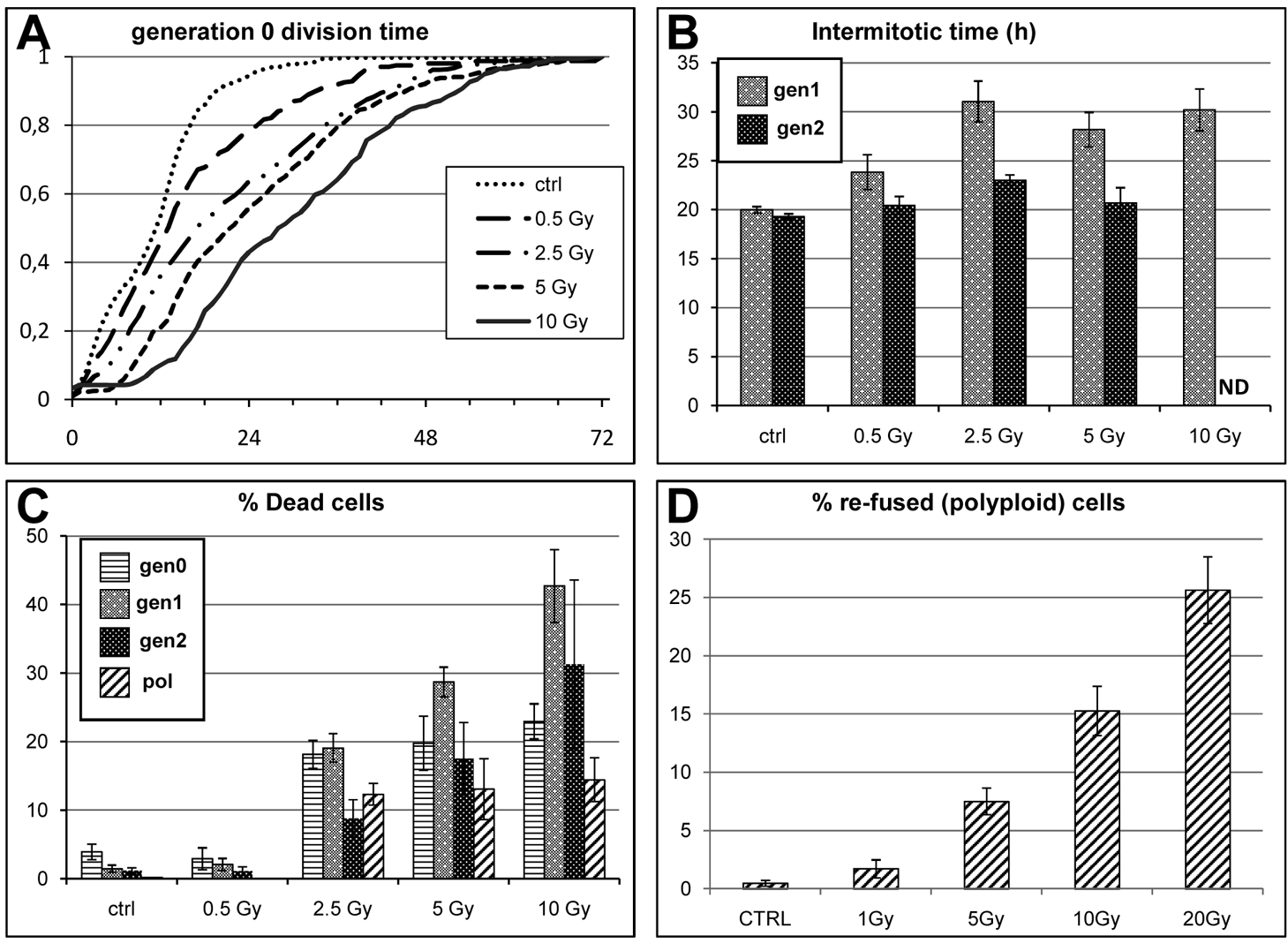

Supplement: Figure S1 — Main statistics of TL experiments. The cumulative distribution of the time to division in gen0 (panel A) and the average Tc in gen1 and gen2 (panel B) were indicative of cell cycle delays, the percentage of dead cells in each generation in the whole 0–72 h observation time (panel C) of the cytolethal effect, the percentage of re-fused cells (panel D) of the polyploidization. Columns and error bars in panels B, C and D represent mean and standard deviation respectively, in at least five independent culture wells. All wells were pooled in panel A. (TIF) [file pcbi.1003293.s004.tif]

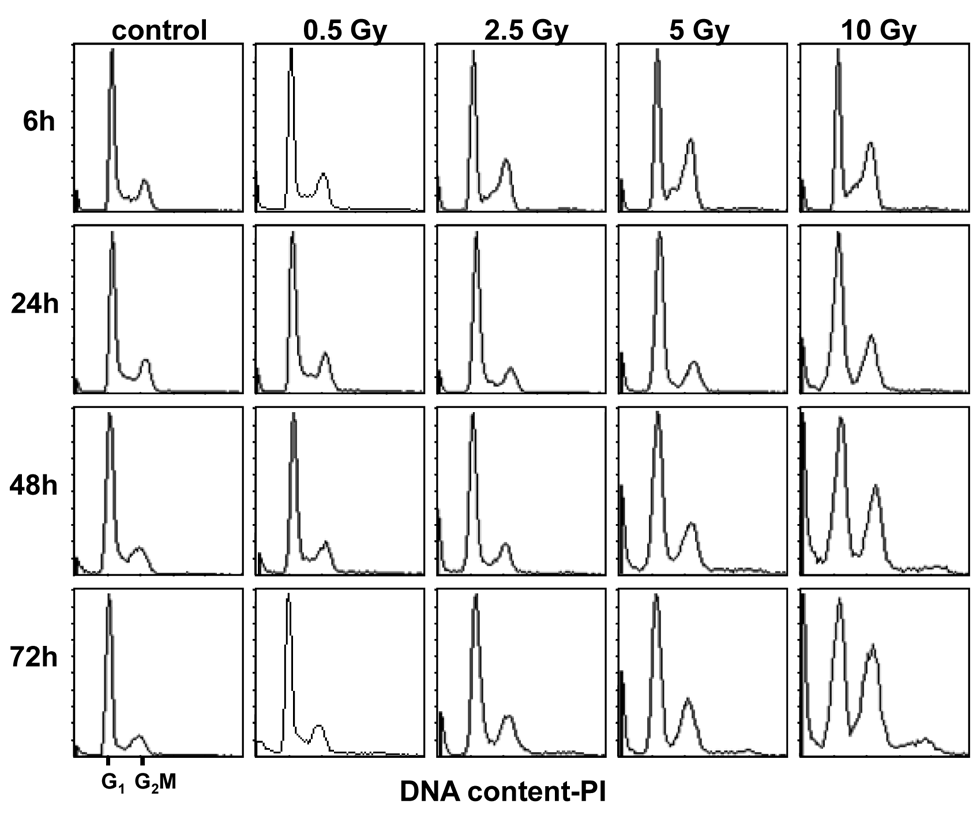

Supplement: Figure S2 — Main results of FC experiments: DNA histograms. Abscissa is proportional to cellular DNA content, with G1 and G2M cells in the positions indicated. Signals below the G1 peak indicate the presence of cell debris, at doses and times consistent with cell death observed with TL. Signals above the G2M peak indicate tetraploid cells, again confirming TL observations. (TIF) [file pcbi.1003293.s005.tif]

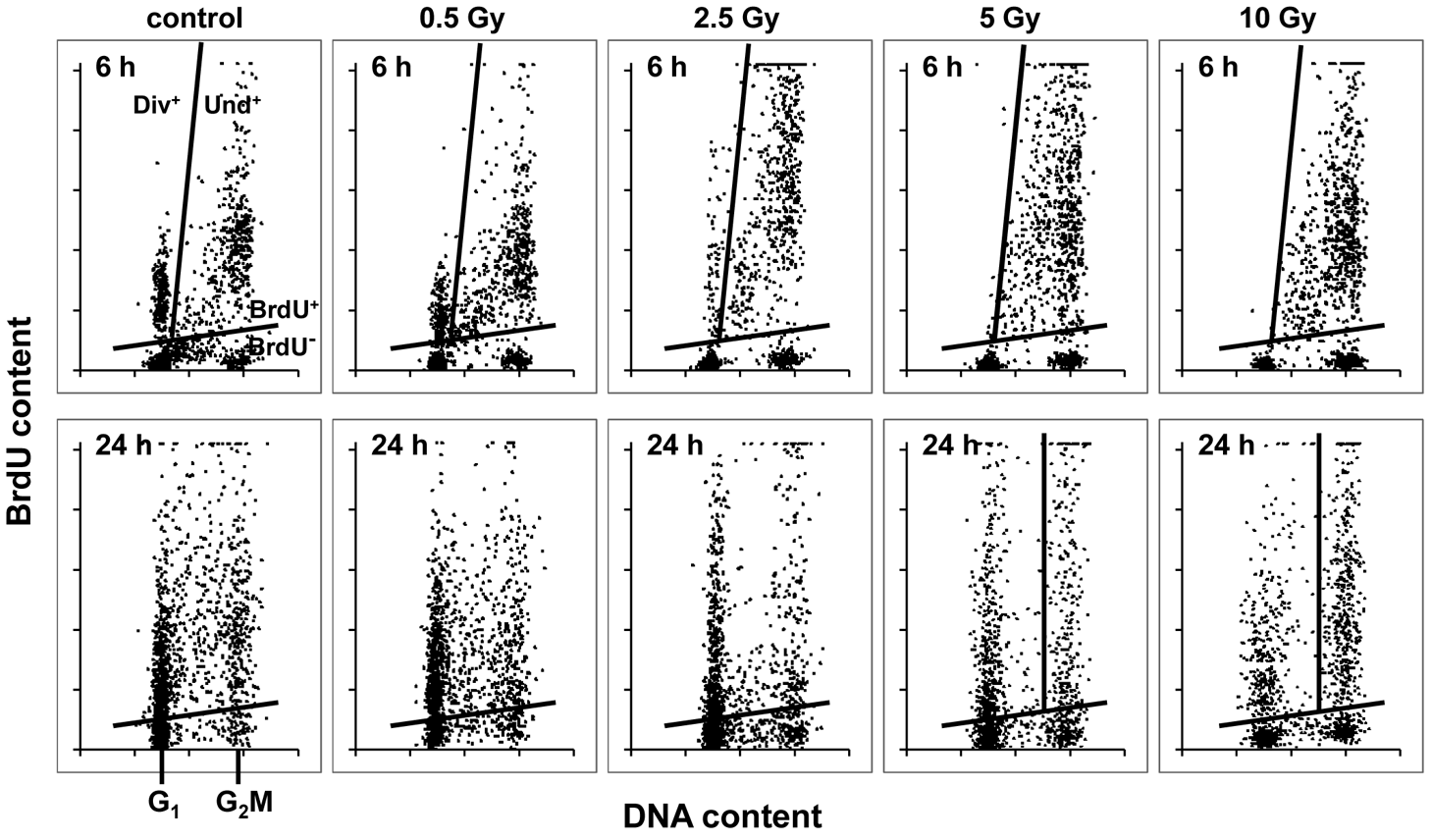

Supplement: Figure S3 — Main results of pulse-chase BrdU experiments. Representative dot plots for a pulse-chase BrdU experiment, taken at 6 h (upper panels) or 24 h (lower panels). Abscissa: cellular DNA content measured by PI fluorescence. The positions of G1 and G2M are indicated. Ordinate: cellular BrdU content measured by Anti-BrdU and a secondary FITC-labeled antibody. The lines mark the region of interest, separating BrdU+ from BrdU− and divided from undivided BrdU+ cell subpopulations. (TIF) [file pcbi.1003293.s006.tif]

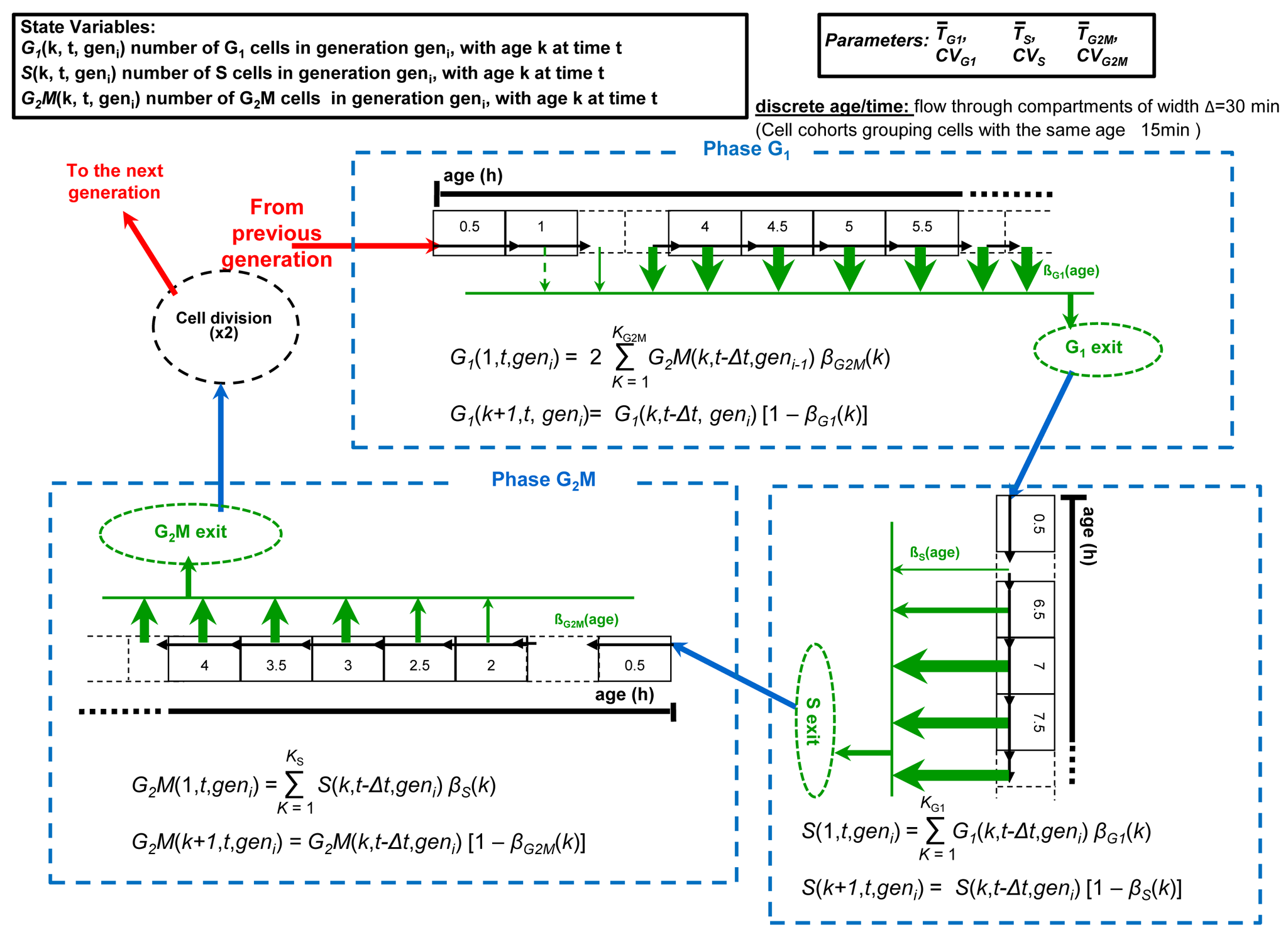

Supplement: Figure S4 — Basic cell cycle model with variable phase durations. Cells enter the first age compartment (0–0.5 h) in a phase “ph” (G1, S or G2M) then gradually progress through the subsequent age compartments, while other cohorts enter the phase. Because the time spent in a phase (Tph) is variable for the cells of the cohort, when the cohort reaches a given age, it has been depleted of the cells that have already completed the phase and a further fraction (βph) of the remaining is expected to exit the phase at that age. The exit probability βph is a function of age that univocally depends on the average () and coefficient of variation (CVph) of the phase durations. At a given time, some cells from all age cohorts complete the phase, collectively forming the pool of exiting cells that will enter the next phase model at the next time. (TIF) [file pcbi.1003293.s007.tif]

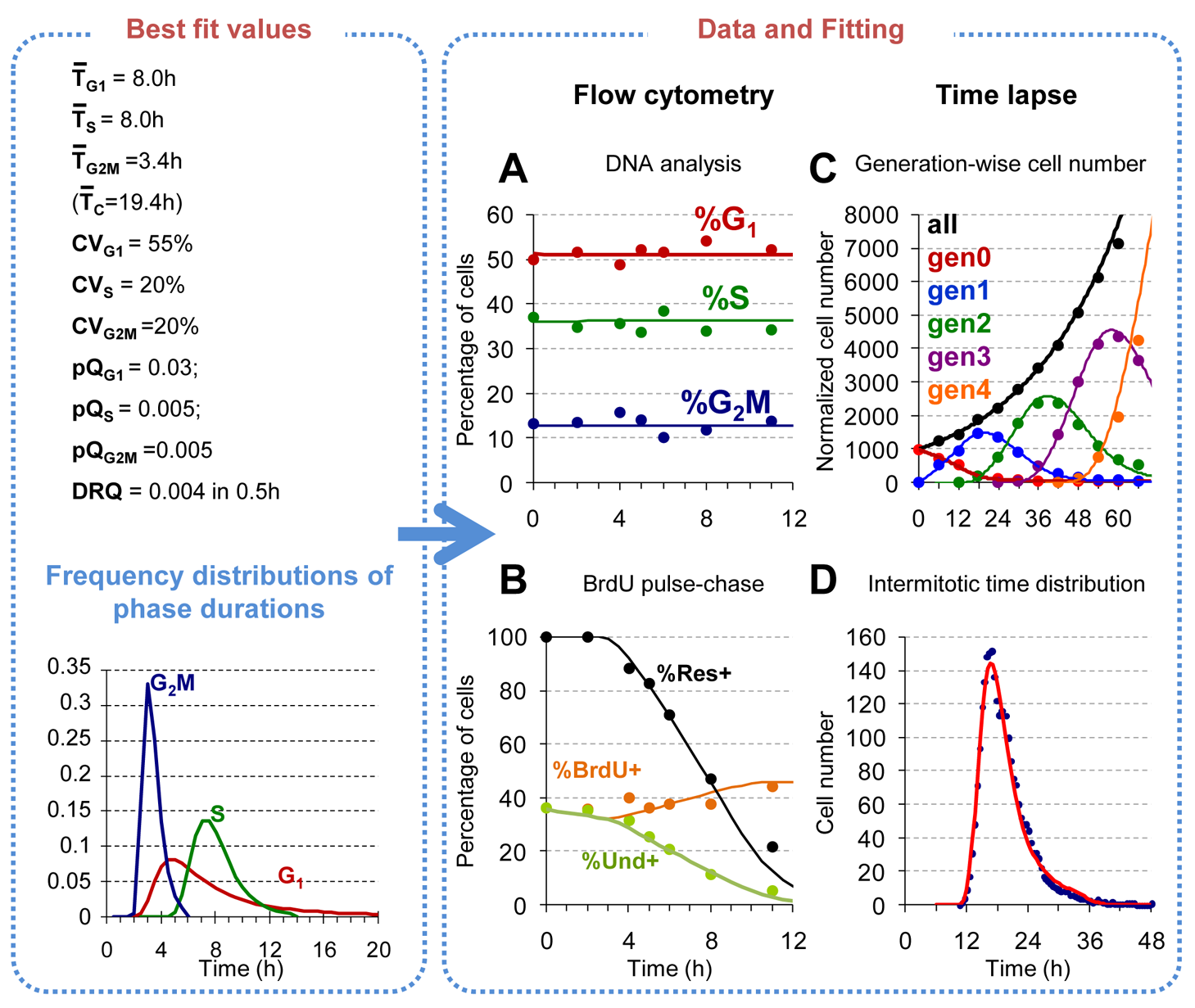

Supplement: Figure S5 — Model of IGROV-1 proliferation (asynchronous growth). Data from preliminary experiments with untreated IGROV-1 cells during exponential growth were fitted with the model described in Computational Methods. Further refinements were included when fitting departed from exponential growth (e.g. the approach to confluence) as reported elsewhere [38] [23]. Left panels: best fit parameters. Main parameters were the average phase durations and their CV (with the frequency distributions of TG1, TS and TG2M shown in the lower panel). Additional parameters (probabilities of quiescence: pQG1, pQS, pQG2M and death rate: DRQ) were included to explain the small percentage (4–8%) of quiescent cells observed by BrdU continuous labelling or TL and spontaneous death (1–3% per generation) observed by TL, as required to fit simultaneously all data shown in the right panels. Right panels: experimental data (symbols) and simulation (continuous lines) including: A) cell cycle percentages from monoparametric FC, B) BrdU+, Und+ and %Res+ (percentage undivided among initially labelled cells, see supplementary Text S1) from FC BrdU pulse chase experiments, C) time course of the number of cells in each generation (normalized assuming N(0) = 1000) from TL, D) frequency distribution of intermitotic times from TL (2480 cells). (TIF) [file pcbi.1003293.s008.tif]

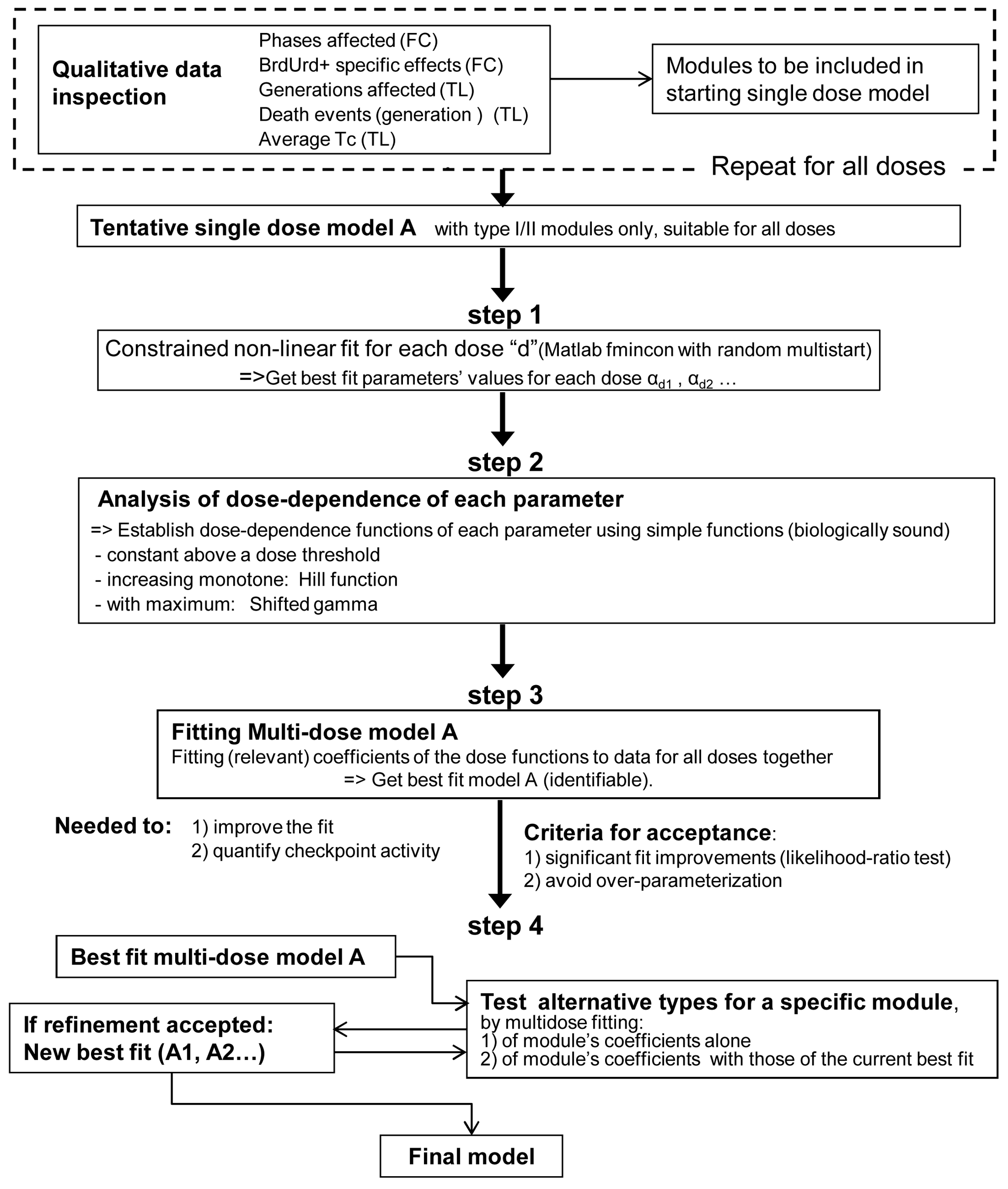

Supplement: Figure S6 — Flow chart of the optimization procedure. (TIF) [file pcbi.1003293.s009.tif]
