# Supplementary material for: Dynamic Rendering of the Heterogeneous Cell Response to Anticancer Treatments
Source: PLoS Comput Biol. 2013 Oct 17;9(10):e1003293. doi: 10.1371/journal.pcbi.1003293 (PMC3798276; doi:10.1371/journal.pcbi.1003293)
Supplement: Table S1 — Qualitative inspection of TL data. (DOC) [file pcbi.1003293.s010.doc]

**Dynamic rendering of the heterogeneous cell response to anticancer treatments**

F. Falcetta, M. Lupi, V. Colombo and P. Ubezio

Table S1. Qualitative inspection of TL data.

|  | delay | Permanent block | Cell death | polyploidization |
| --- | --- | --- | --- | --- |
| Generation 0 | ≥0.5 Gy | ≥5 Gy | ≥2.5 Gy | absent |
| Generation 1 | ≥0.5 Gy | ≥2.5 Gy | ≥2.5 Gy | ≥0.5 Gy |
| Generation 2 | 2.5 Gy | ≥2.5 Gy | ≥2.5 Gy | ≥0.5 Gy |
