# Supplementary material for: Dynamic Rendering of the Heterogeneous Cell Response to Anticancer Treatments
Source: PLoS Comput Biol. 2013 Oct 17;9(10):e1003293. doi: 10.1371/journal.pcbi.1003293 (PMC3798276; doi:10.1371/journal.pcbi.1003293)
Supplement: Table S2 — Modules and parameters of the single-dose model A. (DOC) [file pcbi.1003293.s011.doc]

**Dynamic rendering of the heterogeneous cell response to anticancer treatments**

F. Falcetta, M. Lupi, V. Colombo and P. Ubezio

Table S2. Modules and parameters of the single-dose model A.

| **Generation** | **G_1_ phase** | **S phase** | **G_2_ phase** | **polyploid** |
| --- | --- | --- | --- | --- |
| **gen0 BrdU^-^ cells** | CP type I (Del) CP type II (pBL, DRBL) Cycling cells death (DR) | CP type I (Del) | CP type I (Del) CP type II (pBL, DRBL) |  |
| **gen0 BrdU^+^ cells** |  | CP type I (Del) | CP type I (Del) CP type II (pBL, DRBL) |  |
| **gen1** | CP type I (Del) CP type II (pBL, DRBL) | CP type I (Del) | CP type I (Del) CP type II (pBL, DRBL) | Pol(pPol*,DRpol*) |
| **gen2** | CP type I (Del) CP type II (pBL, DRBL) | CP type I (Del) | CP type I (Del) CP type II (pBL, DRBL) | Pol(pPol*,DRpol*) |

*The same parameter was used for gen1 and gen2, because polyploid cells were pooled irrespective of the generation. CP stands for checkpoint module (see Computational methods in main text).
