# Supplementary material for: Dynamic Rendering of the Heterogeneous Cell Response to Anticancer Treatments
Source: PLoS Comput Biol. 2013 Oct 17;9(10):e1003293. doi: 10.1371/journal.pcbi.1003293 (PMC3798276; doi:10.1371/journal.pcbi.1003293)
Supplement: Table S4 — Dose-dependence and variable coefficients in the final model. (DOC) [file pcbi.1003293.s013.doc]

**Dynamic rendering of the heterogeneous cell response to anticancer treatments**

F. Falcetta, M. Lupi, V. Colombo and P. Ubezio

Table S4. Dose-dependence and variable coefficients in the final model.

| **Module** | **Parameter** | **Function type** | **Dose-response coefficients** | |
| --- | --- | --- | --- | --- |
|  |  |  | **Variable** | **Fixed** |
| G_1_gen0 | pBL | Hill | *max_pBL_, Dm_pBL_* | *ϒ_pBL_=2.5* |
|  | Rec | Hill | *max_Rec_, Dm_Rec_* | *ϒ_Rec_=-1* |
|  | DRBL | constant | *cost*_DRBL_ | Dth_DRBL_=1.5 |
|  | DR | constant | *cost*_DR_ | Dth_DR_=1.5 |
| S_BrdU-_gen0 | Del | constant | *cost_Del_* | Dth_Del_=7.5 |
| S_BrdU+_gen0 | Del | constant |  | *cost_Del_=0.25*  Dth_Del_=1.5 |
| G_2 BrdU-_gen0 | pBL | Hill | *Dm_pBL_* | *max_pBL_=1 ϒ_pBL_=2.5* |
|  | Rec | Hill | *max_Rec_, Dm_Rec_* | *ϒ_Rec_=-2.5* |
|  | DRBL | constant | *cost*_DRBL_ | Dth_DRBL_=1.5 |
| G_2 BrdU+_gen0 | pBL | Hill | *Dm_pBL_* | *max_pBL_=1 ϒ_pBL_=1.5* |
|  | Rec | Hill | *max_Rec_, Dm_Rec_* | *ϒ_Rec_=-1* |
|  | DRBL | constant | *cost*_DRBL_ | Dth_DRBL_=1.5 |
| G_1_gen1 | pBL* | Hill | *max_pBL_,Dm_pBL_* | *ϒ_pBL_=2.5* |
|  | Rec* | Hill | *max_Rec_, Dm_Rec_* | *ϒ_Rec_=-2.5* |
|  | DRBL | Hill | *max*_DRBL_, *Dm*_DRBL_ | *ϒ_DRBL_=10* |
| Sgen1 | Del | Hill | *max_Del_, Dm_Del_* | *ϒ_Del_=2.5* |
| G_2_gen1 | pBL* | Hill | *max_pBL_,Dm_pBL_* | *ϒ_pBL_=2.5* |
|  | Rec | constant | *cost_Rec_* | *Dth_Rec_=7.5* |
|  | DRBL | constant | *cost*_DRBL_ | Dth_DRBL_=7.5 |
| G_1_gen2 | DRBL | Hill | *max*_DRBL_*, Dm*_DRBL_ | *ϒ_DRBL_=10* |
| G_2_gen2 | DRBL | constant | *cost*_DRBL_ | Dth_DRBL_=1.5 |
| pol | pPol | Hill | *max_pPol_* | *Dm*_pPol_=8.5 *ϒ*_pPol_*=1* |
|  | DRpol | Hill | *max*_DRpol_ | *Dm*_DRPol_=1.5 *ϒ*_DRPol_*=2.5* |

*The same parameter was used for gen1 and gen2.
