# Supplementary material for: Dynamic Rendering of the Heterogeneous Cell Response to Anticancer Treatments
Source: PLoS Comput Biol. 2013 Oct 17;9(10):e1003293. doi: 10.1371/journal.pcbi.1003293 (PMC3798276; doi:10.1371/journal.pcbi.1003293)
Supplement: Table S5 — Best fit values of variable parameters in the final model, with 95% confidence intervals. (DOC) [file pcbi.1003293.s014.doc]

**Dynamic rendering of the heterogeneous cell response to anticancer treatments**

F. Falcetta, M. Lupi, V. Colombo and P. Ubezio

Table S5. Best fit values of variable parameters in the final model, with 95% confidence intervals, evaluated either by the Monte Carlo or by the likelihood-based methods (see Uncertainty analysis section in text S4).

| **Module** | **Parameter** | **Function type** | **Variable** | **Best fit** | **M 95% CI^a^** | **L 95% CI^b^** |
| --- | --- | --- | --- | --- | --- | --- |
| G_1_gen0 | pBL | Hill | *max_pBL_* | 0.68 | 0.62 - 0.73 | 0.64-0.72 |
|  |  |  | *Dm_pBL_* | 0.8 | 0.6 - 0.9 | 0.6-1.0 |
|  | Rec | Hill | *max_Rec_* | 0.19 | 0.04 - 0.24 | 0.16-0.24 |
|  |  |  | *Dm_Rec_* | 0.5 | 0.5 - 4.1 | 0.4-0.6 |
|  | DRBL | constant | *cost*_DRBL_ | 0.06 | 0.03 - 0.07 | 0.04-0.08 |
|  | DR | constant | *cost*_DR_ | 0.01 | 0 - 0.02 | 0.01-0.02 |
| S_BrdU-_gen0 | Del | constant | *cost_Del_* | 0.64 | 0.54 - 0.68 | 0.55-0.71 |
| G_2BrdU-_gen0 | pBL | Hill | *Dm_pBL_* | 2.1 | 1.6 - 2.9 | 1.3-2.9 |
|  | Rec | Hill | *max_Rec_* | 0.40 | 0.27 - 0.60 | 0.29-0.62 |
|  |  |  | *Dm_Rec_* | 8.2 | 6.5 - 9.9 | 6.3-12.9 |
|  | DRBL | constant | *cost*_DRBL_ | 0 | 0 - 0.011 | 0-0.006 |
| G_2 BrdU+_  gen0 | pBL | Hill | *Dm_pBL_* | 1.2 | 0.9 - 1.6 | 1.0-1.5 |
|  | Rec | Hill | *max_Rec_* | 0.07 | 0.05 - 0.13 | 0.06-0.08 |
|  |  |  | *Dm_Rec_* | 10.0 | 2.8 – 10.0 | 7.7-13.8 |
|  | DRBL | constant | *cost*_DRBL_ | 0 | 0 - 0.003 | 0-0.001 |
| G_1_gen1 | pBL* | Hill | *max_pBL_* | 0.53 | 0.49 - 0.55 | 0.51-0.55 |
|  |  |  | *Dm_pBL_* | 0.6 | 0.5 - 0.7 | 0.5-0.6 |
|  | Rec* | Hill | *max_Rec_* | 0.03 | 0.02 - 0.05 | 0.04-0.05 |
|  |  |  | *Dm_Rec_* | 1.3 | 0.9 - 1.7 | 1.1-1.5 |
|  | DRBL | Hill | *max*_DRBL_, | 0.015 | 0.013 - 0.017 | 0.014-0.018 |
|  |  |  | *Dm*_DRBL_ | 2.3 | 2.0 - 2.5 | 2.2-2.5 |
| Sgen1 | Del | Hill | *max_Del_,* | 0.54 | 0.49 - 0.57 | 0.52-0.57 |
|  |  |  | *Dm_Del_* | 0.7 | 0.6 - 0.8 | 0.6-0.8 |
| G_2_gen1 | pBL* | Hill | *max_pBL_,* | 0.81 | 0.76 - 0.87 | 0.73-0.90 |
|  |  |  | *Dm_pBL_* | 2.5 | 2.2 - 2.8 | 2.2-2.8 |
|  | Rec | constant | *cost_Rec_* | 0.08 | 0.05 - 0.16 | 0.06-0.13 |
|  | DRBL | constant | *cost*_DRBL_ | 0.07 | 0.03 - 0.19 | 0.04-0.18 |
| G_1_gen2 | DRBL | Hill | *max*_DRBL_*,* | 0.2 | 0.08 - 0.20 | 0.09-0.38 |
|  |  |  | *Dm*_DRBL_ | 6.9 | 6.2 - 7.2 | 6.4-7.7 |
| G_2_gen2 | DRBL | constant | *cost*_DRBL_ | 0.10 | 0.05 - 0.19 | 0.05-0.56 |
| pol | pPol | Hill | *max_pPol_* | 0.84 | 0.78 - 0.90 | 0.79-0.90 |
|  | DRpol | Hill | *max*_DRpol_ | 0.003 | 0.002 - 0.003 | 0.002 - 0.004 |

*The same parameter was used for gen1 and gen2.

^a^ M 95% CI: 95% confidence intervals, based on the Montecarlo method.

^b^ L 95% CI: Likelihood-based 95% confidence intervals.
