# Supplementary material for: Dynamic Rendering of the Heterogeneous Cell Response to Anticancer Treatments
Source: PLoS Comput Biol. 2013 Oct 17;9(10):e1003293. doi: 10.1371/journal.pcbi.1003293 (PMC3798276; doi:10.1371/journal.pcbi.1003293)
Supplement: Text S1 — First-level data analyses. (DOC) [file pcbi.1003293.s015.doc]

**Dynamic rendering of the heterogeneous cell response to anticancer treatments**

F. Falcetta, M. Lupi, V. Colombo and P. Ubezio

**Supporting Text S1: First-level data analyses**

Flow cytometry

The percentages of cells in the cell cycle phases (%G_1_, %S, %G_2_M) were calculated from monoparametric DNA histograms by a modified method of Gaussians [1]. When events in the 4N-8N region (tetraploid cells) were detected above the control baseline, they were pooled with G_2_M, because G_1_ tetraploid were not distinguishable from G_2_M cells on the basis of DNA content. No events >8N were detected.

Biparametric DNA-BrdU plots were analysed setting suitable regions of interest separating BrdU^+^ from BrdU^-^ cells and undivided (Und^+^) from divided (Div^+^) BrdU^+^ cells (see Figure 2A in the main text). The reduced ability of BrdU^+^ cells to complete the cell cycle after treatment was quantified by the percentage of residual BrdU^+^ undivided cells (%Res^+^), i.e. the percentage of cells originally in S-phase that were still undivided at a given time, calculated as

%Res^+^ =100 × %Und^+^ / (%Und^+^ + %Div^+^/2).

All cell cycle percentages (different times and doses) obtained with the two flow cytometric methods were collected in the FC database.

Time-lapse imaging

Analysis of TL lineage data led to computation of the frequency distributions of intermitotic times and cell events (mitosis (M), death (D), survival at 72h (S), re-fusion (R) or loss from the field of view (FL)) and to the time course of the number of cells.

i) Frequency distribution of intermitotic times. For each dividing cell, the intermitotic time (equivalent to the cell cycle duration, Tc) was calculated as the difference between the times of two subsequent divisions. This cannot be calculated for gen0 cells, as they were born at an unknown time before the start of the experiment. In this case, we calculated the frequency distribution of the times corresponding to the first mitosis.

ii) Frequencies of the cell events in each generation (fM_gen(i)_ for cells that divide, fD_gen(i)_ for dead cells, fS_gen(i)_ for surviving cells). These were calculated within the pool of cells remaining in the field of view in each generation, formally assuming that the frequency of the different outcomes in a cell generation was the same within cells remaining or exiting the field of view.

iii) Time course of the number of cells in each generation, relative to the initial cell number (excluding FL cells in gen0). In order to precisely calculate the expansion of the original pool of gen0 cells, we took account of the contribution of FL cells in gen1 and subsequent generations and their descendants (“FL pool”), as follows. With fM_gen(i)_ the previously calculated frequency of mitotic (dividing) cells and NFL_gen(i)_ the observed number of FL cells within gen(i), the overall number of FL cells entering generation “i” from the previous generation “i-1” (NinFL_gen(i)_) was recursively calculated as NinFL_gen(i)_ = 2x (NinFL_gen(i-1)_ + NFL_gen(i-1)_) x fM_gen(i-1)_ and the corNin_gen(i)_ = obsNin_gen(i)_ + NinFL_gen(i)_ where corNin and obsNin are the corrected and observed overall number of cells entering gen(i). Then the corrected time-course was obtained with the formula: corN(t)_gen(i)_ = obsNR(t)_gen(i)_ x corNin_gen(i)_ / (obsNin_gen(i)_ - NFL_gen(i)_) i.e. assuming that the corrected time course was the same as the observed time course within cells remaining in the field of view (NR_gen(i)_(t)), multiplied by a constant factor (the ratio of the corrected overall number to observed number of cells not classified as FL).

### Microsoft Excel routines to calculate the number of cells present at each time from a lineage database are available from the corresponding author.

References

1. Ubezio P (1985) Microcomputer experience in analysis of flow cytometric DNA distributions. Comput Programs Biomed 19: 159-166.
