# Supplementary material for: Dynamic Rendering of the Heterogeneous Cell Response to Anticancer Treatments
Source: PLoS Comput Biol. 2013 Oct 17;9(10):e1003293. doi: 10.1371/journal.pcbi.1003293 (PMC3798276; doi:10.1371/journal.pcbi.1003293)
Supplement: Text S2 — Data inspection. (DOC) [file pcbi.1003293.s016.doc]

**Dynamic rendering of the heterogeneous cell response to anticancer treatments**

F. Falcetta, M. Lupi, V. Colombo and P. Ubezio

**Supporting Text S2: Data inspection**

We are able to give a preliminary view of the effects of X-ray exposure just looking at the data generated by the first level of analysis of flow cytometry and time-lapse experiments. By time-lapse microscopy, the antiproliferative effect is viewed from a generation-wise perspective, and the three lineage examples reported in Figure 2B of the main text, referring to cells exposed to 5 Gy, already qualitatively illustrate the appreciate cytostatic and cytotoxic effects in the lineages of irradiated cells.

These examples indicated that cells adopted different responses to radiation, some unaffected, others delayed, others unable to divide up to the end of the observation, others re-fusing, others dying. Different types of cell death were observed in the irradiated cell populations. Clear apoptotic hallmarks were observed in some instances (blebbing, shrinkage, extrusion of apoptotic bodies), while in other cases cells suddenly inflated, resulting in a big, inactive spherical bubble. In other circumstances, cells remained small and round without any active movement up to the end of the observation and were classified as dead. Distinction of the various types of death was beyond the aim of the present study.

To appreciate the real impact of the different outcomes on the whole population, avoiding over-emphasis only on episodic phenomena, we had to follow a representative number of lineages for each dose, to collect the data in a lineage database and to obtain statistics of the results. The following number of lineages and cells were included in this database:

|  | lineages | cells |
| --- | --- | --- |
| control | 343 | 5626 |
| 0.5 Gy | 306 | 4301 |
| 2.5 Gy | 538 | 2342 |
| 5 Gy | 474 | 1646 |
| 10 Gy | 494 | 1192 |

Cell population statistics are shown in Figure S1. Panel a indicates that cell division of gen0 cells is delayed in a dose-dependent way. Delays of cells that eventually divide is indicated by the average intermitotic time shown in panel b. T̅_C_ increase from 0.5 Gy in gen1, with a plateau at 2.5 Gy, while minor delays were detected in gen2, especially with 2.5 Gy. The frequencies of different outcomes are shown in panels c-d. In gen0, the majority of cells were able to divide even at the highest dose. No cell death above the control level was observed with 0.5 Gy in any generation. At higher doses, cell death was present and increased with dose (c). Similarly, re-fusion events were dose-dependent, in both gen1 and gen2 (d). In these two generations, around 25% of “surviving” cells were observed with 2.5 Gy and higher doses, indicating definitely arrested cells (not shown). Table S1 summarizes the results: cell cycle delays were present even at 0.5 Gy, while cell death was detectable from 2.5 Gy, and up to the gen2 cells, and some sibling cells eventually re-fused, producing a subset of polyploid cells within which mitosis was rare.

Flow cytometry data provided complementary information, suggesting the activation of blocks/delays in all cell cycle phases, but without distinguishing different generations.

Figure S2 shows DNA histograms of control and irradiated samples at different times and the cell cycle phase percentages. Simple visual inspection of the histograms did not show up differences between control and the 0.5 Gy-irradiated samples. At higher doses there was a partial accumulation of cells in G_2_M 6h after irradiation, with decreases in the percentages of G_1_ and S cells. At 24h %G_2_M cells diminished, but without reaching control values, while %G_1_ increased and %S remained low. These results suggested that both G_1_ and G_2_M checkpoints were active. At 48 and 72h the %G_2_M in treated samples was still higher than control, whereas %S was lower.

Short-term effects were investigated with a pulse-chase BrdU experiment. The dot plots in Figure S3 show that most BrdU^+^ cells irradiated with 2.5 Gy or higher doses were unable to divide and reach gen1 at 6h, but accumulated undivided in G_2_M, while a much larger proportion of control and 0.5 Gy irradiated BrdU^+^ cells divided and reached the next G_1_ phase. Similarly, most BrdU^-^ irradiated cells were unable to exit G_1_, while a considerable proportion of control BrdU^-^ cells was detected in S phase. Thus there is co-activation of both G_1_ and G_2_M checkpoints, resulting in the block of irradiated cells in the respective phases, immediately after radiation. Comparison of the movements of the cloud of undivided BrdU^+^ cells through S phase in the dot plots of control and irradiated samples revealed a delay also in crossing S phase, indicating activation of the S checkpoint.

The pulse-chase experiment was extended to 24h but data analysis was limited to the highest doses (5 and 10 Gy), where the majority of BrdU+ cells were still undivided at that time, together with a group of divided cells, almost all in G_1_. This indicated a persistent G_2_M block, followed by a G_1_ block for cells that eventually divided. At lower doses, the presence of cycling gen1 cells overlapped and mixed with undivided gen0, preventing any reliable measure of residual undivided cells.
