# Supplementary material for: Dynamic Rendering of the Heterogeneous Cell Response to Anticancer Treatments
Source: PLoS Comput Biol. 2013 Oct 17;9(10):e1003293. doi: 10.1371/journal.pcbi.1003293 (PMC3798276; doi:10.1371/journal.pcbi.1003293)
Supplement: Text S3 — Modelling proliferation of untreated cells. (DOC) [file pcbi.1003293.s017.doc]

**Dynamic rendering of the heterogeneous cell response to anticancer treatments**

F. Falcetta, M. Lupi, V. Colombo and P. Ubezio

**Supporting Text S3: Basic cell cycle modeling**

This supplementary summarizes the concepts of the basic cell cycle routine in the core of program. These have been explored in previous studies of our group with single-cycle models, where newborn cells (from mitosis of a mother cell) re-enter in the same cell cycle of the cells of the previous generations (single-cycle models) [1-3]. In the present version of our modelling approach, multiple cell cycle routines are connected, transferring the offsprings of the cells ending a cycle (representing a single generation) to the beginning of a subsequent cell cycle (the next generation) and so on (multi-generation models).

The basic cell cycle model (Figure S4) reproduces the flow of cells through the cycle, acknowledging that individual cells spend different times in the same phase by introducing probability distributions *F_ph_(k_ph_)*, defined as the probability that a cell ends a phase *ph* (either G_1_, S or G_2_M) in a time k_ph_ Δt, being Δt the adopted time step (see below). *F_G1_(k_G1_)*, *F_S_(k_S_)* and *F_G2M_(k_G2M_)* were assumed to be independent of each other and calculated on the basis of either Gaussian (G(k)), lognormal (L(k)) or reciprocal-normal (R(k)) functions:

$$G\left(k\right)=A\cdot e^{-\frac{1}{2}\frac{\left(k\cdot\Delta t-a\right)^{2}}{b^{2}}}$$

$$L\left(k\right)=\frac{A}{k\cdot\Delta t}\cdot e^{-\frac{1}{2}\frac{\left(\ln\left(k\cdot\Delta t\right)-a\right)^{2}}{b^{2}}}$$

$$R\left(k\right)=\frac{A}{\left(k\cdot\Delta t\right)^{2}}\cdot e^{-\frac{1}{2}\frac{\left(\frac{1}{k\cdot\Delta t}-a\right)^{2}}{b^{2}}}$$

where the parameters “a” and “b” are connected to the average and coefficient of variation (CV) of phase duration and A is the normalizing constant. The selected functions were truncated at an appropriately long age K_ph_Δt (i.e. K_ph_ is the last compartment in a phase) and normalised.

The reciprocal-normal distribution, equivalent to normally distributed cycling “rates”, was already proposed by Sisken and Morasca [4]. Castor [5] studied overall cell cycle time distributions from several published papers with a two-phases (G_1_ and SG_2_M) model, demonstrating that a reciprocal-normal distribution of T_G1_ enabled to obtain proper fits in all instances, without requiring a random transition between the two “phases”, as proposed by the classical Smith and Martin paper [6]. Notice that the Smith and Martin model, not considering additional intercellular variability of the phases, predicts a shifted, exponentially decreasing is distribution, not consistent with the observed broadened distribution of Tc. Our experience is in the same line, and our experimental distribution of Tc (supplementary Figure S5, bottom right panel) is better fitted by a reciprocal-normal than by other distributions (not shown).

Thus, the reciprocal-normal appeared to us a natural choice for the distribution also of the individual phases, although a direct measure of each of them is not possible. A posteriori, this choice was successful to fit at the same time different kinds FC and TL data (Figure S5). Nevertheless, our program enables the user to select log-normal as alternative to reciprocal-normal distributions. The choice of log-normal led to similar fittings than the reciprocal-normal distributions (not shown), suggesting that data were sensitive to mean and standard deviation, but less sensitive to the shape of the distribution. In principle, even more complex distributions could be adopted, or combinations including both a transition probability and distributed durations of phases, but the solution would not be univocal.

Independence of G_1_, S and G_2_M phase durations was assumed in the absence of evidence of precise relationships between these durations, at least in cancer cells. If such relationships would exist, other kinds of data should be taken into consideration.

From *F_ph_(T_ph_)* we calculate the $\beta_{ph}\left(k\right)$ factor (exit probability), defined as:

$\beta_{ph}\left(k\right)=\frac{F_{ph}\left(k\right)}{1-\sum_{j=1}^{k-1} F_{ph}\left(j\right)}$ which gives the proportion of cells completing a phase at age k, among the cells that reach age k-1. A cohort of cells enters the first age compartment (0-0.5h) in a phase (e.g. G_1_) then progresses through the subsequent age compartments, while other cohorts enter G_1_. Because the time spent in G_1_ (T_G1_) is variable for the cells of the cohort, when the cohort reaches a given age k, it has been depleted of the cells that have already completed G_1_ and a further fraction β_G1_(k) of the remaining is expected to exit G_1_ at that age. At a given time, some cells from all age cohorts complete G_1_, collectively forming the pool of G_1_ exiting cells that will enter the S-phase model at the next time.

Using the dynamics is described by the balance equations shown in Figure S4, defined by the choice of the three $F_{ph}\left(k\right)$ distributions, completely described by six parameters: {T̅_G1_, CV_G1_, T̅_S_, CV_S_, T̅_G2M_, CV_G2M_}, i.e. the average and coefficient of variation of the phase durations.

The adopted time step in the present study was 0.5h (2.5% of average Tc), which is a small interval with respect to the time variations of the data. A shorter time-step (0.1 h) was evaluated for the simulation of IGROV-1 cell proliferation, increasing the time machine (about forty times for a multi-dose simulation) with negligible changes of the simulated results (< 0.3% difference in both FC percentages and TL number of cells in each generation). A somewhat higher difference was found in the multi-dose simulation of treatments (<1% in FC percentages and < 1.5% in TL simulated data), but still lower than experimental precision of these kinds of data. Thus the 0.5 h appeared a reasonable tradeoff between precision of the simulation and reduction of the time-machine, especially for fitting purposes.

This model can be further generalised, including a “G0-like” subphase (or “A-state”) within G_1_ and characterized in kinetics terms by a stochastic output, as postulated by transition probability models [6] [7], irrespective of functional identification, as a G_1a_ or G0 phase, which depends on the cell population of interest. This option was explored in early “single-cycle” versions of our program [8] but was never found necessary in subsequent studies with tumour cells in vitro. We expect that in *in vivo* modelling of tumor expansion, dealing with phenomena lasting weeks or months (i.e. different time scales respect to those typical of drug testing in vitro) the G0 phase could be considered, possibly with G0 mean transit time much longer than Tc (i.e. the sum of G_1_, S and G_2_M). Moreover, typical experiments in the immunological field, aimed to evaluate the recruitment into proliferation of G0 lymphocytes upon suitable stimuli, could require this additional phase. For these reasons, the G0 option was maintained also in the new versions of our model to enable users to consider G0 when required by specific cell systems and it is included in the executable files accompanying this paper.

References

1. Montalenti F, Sena G, Cappella P, Ubezio P (1998) Simulating cancer-cell kinetics after drug treatment: Application to cisplatin on ovarian carcinoma. Physical Review E 57: 5877-5887.

2. Sena G, Onado C, Cappella P, Montalenti F, Ubezio P (1999) Measuring the complexity of cell cycle arrest and killing of drugs: kinetics of phase-specific effects induced by taxol. Cytometry 37: 113-124.

3. Ubezio P (2004) Unraveling the complexity of cell cycle effects of anticancer drugs in cell populations. Discrete and Continuous Dynamical Systems - Series B (DCDS-B) 4: 323 - 335.

4. Sisken JE, Morasca L, Kibby S (1965) Effects of temperature on the kinetics of the mitotic cycle of mammalian cells in culture. Exp Cell Res 39: 103-116.

5. Castor LN (1980) A G1 rate model accounts for cell-cycle kinetics attributed to 'transition probability'. Nature 287: 857-859.

6. Smith JA, Martin L (1973) Do cells cycle? Proc Natl Acad Sci U S A 70: 1263-1267.

7. Burns FJ, Tannock IF (1970) On the existence of a G 0 -phase in the cell cycle. Cell Tissue Kinet 3: 321-334.

8. Ubezio P (1993) Relationship between flow cytometric data and kinetic parameters. Eur J Histochem 37/suppl. 4: 15–28.
