# Supplementary material for: Dynamic Rendering of the Heterogeneous Cell Response to Anticancer Treatments
Source: PLoS Comput Biol. 2013 Oct 17;9(10):e1003293. doi: 10.1371/journal.pcbi.1003293 (PMC3798276; doi:10.1371/journal.pcbi.1003293)
Supplement: Text S4 — Optimization procedure and softwares. (DOC) [file pcbi.1003293.s018.doc]

**Dynamic rendering of the heterogeneous cell response to anticancer treatments**

F. Falcetta, M. Lupi, V. Colombo and P. Ubezio

**Supporting Text S4: Optimization procedure and softwares**

Optimization procedure

Trial-and-error simulations used in previous studies formally allow “falsifying” (i.e. excluding) proliferation scenarios inconsistent with the data, reaching a selection of the representative scenarios of the phenomena in play, where the effects on cells of different generations were mixed and only approximately separated by their time-dependence [1-5]. This procedure has been completely revised, with the aim of reaching a realistic rendering of the proliferation, robustly consistent with a wide data set, properly catching not only the flow of the cells through the cell cycle but also their passage through subsequent generations. This was achieved by including time-lapse data and adopting a formal optimization procedure using a protocol to guide the choice of the model (intended as the ensemble of the modules required to describe the data), and powerful non-linear fitting techniques. These enabled us to obtain the set of parameter values providing the best fit of the data for a given model, and to evaluate its uniqueness. The need to fit data from different platforms (i.e. flow cytometry and time lapse imaging) at the same time, with different measurements units and precision required a preliminary study to devise a suitable objective function (see below). It is essentially a likelihood function, which also permits an estimation of likelihood-based confidence intervals with standard statistics.

The flow chart shown in Figure S6 summarizes the whole optimization process, which proceeded through four steps to achieve a robust rendering of the proliferation for all doses together:

1. Definition of the modules required and data fitting of individual doses
2. Analysis of dose-dependence of single parameters
3. Multi-dose fit
4. Refinement of modules

The procedure is detailed below, as it was applied to fit the data of the experiments presented in this work.

*Step 1. Definition of the modules required and data fitting of individual doses*

In the first step of the optimization procedure, we independently fitted the data for each dose of radiation with a relatively simple model (Model A). Model A was drawn on the basis of qualitative data inspection (supplementary Text S2) and included type I modules to render the activity of S phase checkpoints and type II joined to type I modules for G_1_ and G_2_M checkpoints, up to gen2. Because some cell death was observed immediately after irradiation with 5 Gy and higher doses, a cycling-cells death module was introduced in G_1_ in gen0. In addition, to consider the possibility of different responses from BrdU^+^ and BrdU^-^ cells, we considered distinct modules for these two cell subsets in gen0. All other parameters were equal within BrdU^+^ and BrdU^-^ cells. Distinct BrdU^+^ and BrdU^-^ modules for the other generations and the introduction of cycling-cells death modules for the other phases of gen0 did not improve the fitting. As concerns delays, qualitative data inspection did not lead us to exclude them in any phase, and similarly the possibility of both G_1_ and G_2_M blocks was accepted in all generations. Therefore the generic model A was drawn with the modules and 28 parameters as reported in Table S2.

*Del_S_* in BrdU^+^ gen0 cells was fixed and calculated by inverting eq. 1, using the values of T̅_S_ and
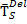
 deduced from 6h DNA-BrdU histograms [6]. The model enabled us to calculate
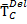
 of dividing cells in gen1 and gen2 based on the values of Del_G1_, Del_S_ and Del_G2M_ (eq. 2), and to compare it with the corresponding experimental measure. Thus, the values of Del_G1_, Del_S_ and Del_G2M_ were constrained to give a
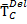
 consistent with the measured T̅_C_ with 15% tolerance (consistent with the error of the measure).

The resulting Model A was fitted to the data set of flow cytometric and time-lapse data for each dose, maximizing the objective function. The fitting was repeated 100 times from random starting values of the parameters, obtaining a pool of scenarios (i.e. a set of “best fit” values) from which we selected the one with the highest likelihood (Scenario α). We examined the pool for scenarios alternative to α that fitted the data with similar performance (not significantly worse in a likelihood ratio test at p=0.05) [7] and were truly distinct from α, i.e. having at least one parameter value significantly different from the one in Scenario α (with no overlapping likelihood-based confidence intervals at p=0.05). We found one (2.5 Gy and 10 Gy), two (0.5 Gy) and three (5 Gy) alternative scenarios to α, named β, γ, δ with decreasing likelihood, suggesting alternative values for specific single modules.

*Step 2. Dose-dependence of single parameters*

The non-uniqueness of the single-dose fitting was solved by imposing dose-response regularities and eventually building a multi-dose model. During the multi-dose fitting each parameter value was constrained to follow specific dose-response functions, greatly reducing the risk of over-fitting. This is like validating the data for one dose using the data for all the others.

We also assumed that the dose-response of each parameter (delay, block or death rates) regularly behaved, entering one of three categories:

1. Constant with a dose threshold, chosen when the best fit parameter values were zero at the lower doses and did not vary at the higher ones. Thus a threshold activating dose (*Dth*) exists and was conventionally assumed as half the concentration between the highest dose with the related parameter equal to zero and the next dose that was experimentally tested.

*Par(D)* = *Cost_par_* for D ≥ *Dth* (or D ≤ *Dth* for recycling parameters)

1. Monotone. Parameters expected to vary monotonically with the dose (e.g. a block or a death parameter, expected to increase with the dose, or recycling, expected to decrease) were fitted with a Hill function:

$$Par\left(D\right)=\frac{\max_{par}\cdot D^{\gamma par}}{D^{\gamma par}+D_{mpar}^{\gamma par}}$$

where *max_par_*, *D_mpar_*, *ϒ_par_*, coefficients represent the maximum, the half maximum effect dose and the sigmoidicity (*ϒ_par_* > 0 for parameters increasing with dose, <0 for decreasing ones).

1. Not monotone, when a parameter value increased at lower doses and diminished at higher ones. Typically this was expected for delay parameters that decreased at higher doses on account of the competition of an irreversible block in the same phase. In this case the dose-dependence was fitted by a shifted gamma distribution:

$$Par\left(D\right)=\max_{par}\left(D-D\min_{par}\right)\cdot e^{\frac{1-\frac{D-D\min_{par}}{\vartheta par}}{\vartheta par}}$$

*Par(D)*=0 when D <=*D_minpar_*.

where *max_par_*, *D_minpar_*, *ϑ_par_* coefficients represent the maximum value, the threshold dose, and the width of the curve.

To reach a first estimate of the coefficients of the dose-response function for each parameter, we started from values in the single-dose best fit scenarios (α, β, γ, δ) selected in step 1 at the different doses (*Par(0.5)*, *Par(2.5)*, *Par(5)* and *Par(10)*), and fitted these values with the Hill or gamma functions. In this way we analysed the dose-dependence of each parameter, selecting the appropriate function.

*Step 3. Multi-dose model and fitting*

The 28 dose-response functions (constant, Hill or gamma) obtained in step 2 were embedded in a version of our simulation program that runs all doses simultaneously using the whole time-lapse and flow-cytometry databases. In this multi-dose model, the coefficients of the dose-response functions become the new variables to be fitted, minimizing a global merit function which is the sum of the –log likelihood of the fits for each dose. The multi-dose starting values of each coefficient were those emerging from the step 2 analysis.

For the multi-dose fit we optimized the 40 internal coefficients of the functions selected as variables in the previous step (Table S2). Some of the coefficients, to which the data were poorly sensitive, were kept fixed in this step, reserving their revision, if necessary, to the subsequent refinement step 4 (see below). As concerns Hill functions, the data were poorly sensitive to changes in sigmoidicity over wide ranges, so *ϒ_par_*s were kept fixed either to the value of 10 for parameters rapidly changing with the dose, to 2.5 for intermediate, or to 1 for parameters changing slowly with the dose. Similarly, for gamma functions, *D_minpar_*, was kept fixed. These coefficients can be revised later during the refinement step 4 (see below).

The 37 variable parameters (Table S3) were optimized in Matlab with the fmincon routine. The procedure converged to the same minimum (multi-dose scenario A) in 70 out of 100 optimization runs with random starting values, the remainder converging to local minima, with significantly worse fitting (likelihood-ratio test). The multi-dose scenario A reproduced the time course of the number of cells in each generation reasonably well, while other data were not satisfactorily fitted at any dose. In addition, model A did not consider the dynamics of re-cycling from block, so it can be used only as an intermediate step towards more realistic modelling of the checkpoint dynamics.

*Step 4. Refinement of modules*

Starting from the multi-dose scenario A, G_1_ and G_2_M checkpoint modules type I and II were progressively replaced by type III for each generation and multi-dose fits were run. When a module was considered for refinement, all the coefficients of the module were fitted, keeping all coefficients of the other modules fixed, and then only the coefficients selected as variables (see step 2) were optimized together with the variables of all modules of the model. The new model was accepted, becoming the reference best fit, if there was significant improvement of the fit, based on the likelihood-ratio test. The procedure can be repeated to reach convergence for the new model.

Because the results at this stage indicated that S-delay, G_1_ and G_2_M block probabilities, and G_1_ recycling in gen1 and gen2 were the same, gen2 coefficients of the dose-response of these parameters were constrained to be equal to the gen1 coefficients, reducing the number of parameters without worsening the fit.

In the last step, gen0 G_1_ and G_2_M checkpoint modules type III were replaced by type IV, with the same procedure. The final model included 32 variable coefficients, reported in Table S4.

The fitting procedure was repeated with 150 random starting values of the variables, 142 converging to the same scenario and the remaining reaching local minima with significantly higher (likelihood ratio test) values of the objective function. Figure 4 in main text shows the fit of the final model to the FC and TL data. Table S5 reports the best fit values of all variable parameters.

*Objective function*

The objective function to be minimized during the optimization procedures is the overall negative log-likelihood, obtained by summing up the contributions of all experimental procedures considered and assuming normally distributed errors with variance typical of each experimental procedure:

The contribution of TL experiments were:

-log(L_Ngen0_) = 0.5 * LN(2πσ_TL_^2^)+∑_t_(Ngen0_model_ (t) - Ngen0_data_ (t))2/(2σ_TL_^2^)

-log(L_Ngen1_) = 0.5 * LN(2πσ_TL_^2^)+∑_t_(Ngen1_model_ (t) – Ngen1_data_ (t))2/(2σ_TL_^2^)

-log(L_Ngen2_) = 0.5 * LN(2πσ_TL_^2^)+∑_t_(Ngen2_model_ (t) – Ngen1_data_ (t))2/(2σ_TL_^2^)

-log(L_Ngen3_) = 0.5 * LN(2πσ_TL_^2^)+∑_t_(Ngen3_model_ (t) – Ngen3_data_ (t))2/(2σ_TL_^2^)

-log(L_Npol_) = 0.5 * LN(2πσ_TL_^2^)+∑_t_(Npol_model_ (t) – Npol_data_ (t))2/(2σ_TL_^2^)

where Ngen0(t), Ngen1(t), Ngen2(t), Ngen3(t) and Npol (t) are the numbers of cells respectively in generations 0, 1, 2, 3 or higher, polyploid at time t, either measured by time lapse (Ngen0_data_ etc.) or predicted by the model (Ngen0_model_ etc.). The numbers of cells in each generation were rescaled to set the maximum to 100 and have homogeneous data. σ_TL_ is a measure of the precision of the Ngen0_data_ etc. and was set at 5 in the same rescaled units. The summation includes the times 0, 6, 12, 24, 30, 36, 42, 48, 54, 60, 66, and 72h (plus the times 1, 2, 3, 4, and 5h in gen0), but the time courses of each generation were considered as a single “data point”, because single time points were not independent.

- log(L_%death72_) = 0.5 *n_death_ * LN(2πσ_death_^2^)+∑_gen_(%death_model_ (gen) - %death_data_ (gen) )2/(2σ_death_^2^)

where %death(gen) is the percentage of cells dead in a given generation between 0 and 72h; the summation includes gen0, gen1, gen2, gen3 (pooled with higher-order generations) and polyploid cells as an additional “generation”; n_death_ is the number of generations (n_death_ = 5); and σ_death_ = 3.

The contributions of FC experiments were:

-log(L_%G1SG2M_) = 0.5*n_%G1SG2M_*LN(2πσ_%G1SG2M_^2^) + ∑_t_((%G_1model_(t) - %G_1 data_(t))^2^ + (%S_model_(t) - %S _data_(t))^2^

+ (%G_2_M_model_ (t) - %G_2_M_data_(t))^2^) / (2σ_%G1SG2M_^2^)

where %G_1_(t), %S(t) and %G_2_M(t) are cell cycle percentages measured by DNA flow cytometry at time t (0, 6, 24, 48, 72h), n_%G1SG2M_ to the number of time points (n_%G1SG2M_ = 5) and σ_%G1SG2M_ = 3.

-log(L_%Res+_) = 0.5*n_%Res+_*LN(2πσ_%Res+_^2^) + ∑_t_(%Res^+^_model_(t) – %Res^+^_data_(t))^2^ / (2σ_%Res+_^2^)

where %Res^+^(t) is the percentage (see first-level data analysis in supplementary Text S1) of residual undivided BrdU^+^ cells at time t, measured by biparametric DNA-BrdU flow cytometry in the pulse-chase BrdU experiments at time t (0, 6, 24h), and σ_%Res+_ = 5. For controls and lower doses %Res^+^_data_(24h) could not be confidently calculated because of the overlap of residual gen0 with gen1 cells in the last phases of the cycle and was omitted.

Thus the objective function minimized in single-dose “d” fittings was

-log(L_d_)=-log(L_Ngen0_)-log(L_Ngen1_)-log(L_Ngen2_)-log(L_Ngen3_)-log(L_Ngen4+_)-log(L_Npol_)-log(L_%death72_) -log(L_%G1SG2M_)-log(L_%Res+_)

In multi-dose fitting the overall objective function was the sum of -log(L_d_) of all doses:

-log(L_MD_) = -∑_d_ log(L_d_)

Uncertainty analysis

To estimate the uncertainty of the parameters of the best fit obtained with the final model we adopted a Mote Carlo procedure [8-10], which was found suitable with complex nonlinear models in different fields [11-13]. For this purpose, we created 1000 entirely synthetic data sets, adding a random normally distributed noise to each simulated datum calculated by the best fit model. The standard error of the noise was given by the root mean square of residuals (difference between experimental and simulated data) for each type, as detailed below. Each synthetic dataset was fit using initial conditions from the best fit of the actual data, producing a set of optimized parameters and calculating all derived quantities, like percentages of blocked cells in each generation. Collecting the results of the fits of all synthetic data sets, we obtained a 1000 elements distribution for each parameter or derived quantity, from which a 95% confidence interval was calculated by ordering the results and keeping the 2.5 and 97.5 percentiles as limits of the interval.

Two different procedures were adopted to evaluate the standard error of the noise for FC and TL data respectively. For cell cycle percentages, random noise was generated independently for %G_1_, %S and %G_2_M at a given time, with standard error given by the root mean square difference between experimental and simulated cell cycle percentages of all times and doses. Then synthetic percentages were equally rescaled to satisfy the constraint %G_1_+%S+%G_2_M = 100. In the case of TL data, like the time course of the number of cells in a generation, the subsequent time points are not independent and the whole profile moves in response to a variation of a parameter value. In order to produce synthetic data for the number of cells in a given generation we first generated a synthetic datum corresponding to time when the number of cells was highest. Then, the ratio between the synthetic and the corresponding best fit simulated datum was calculated and used as rescaling factor for the whole time course.

We considered also a different approach to the estimate of the uncertainty of the best fit value of parameters, calculating likelihood-based confidence intervals, giving the range of each parameter within which the fit remains not significantly worse than that obtained with the best fit parameter values. The likelihood ratio test statistics (LRTS) was used to compare a fit with a given set of parameters “X” with the best one (LRTS = 2(log(L_best_) - log(L_X_)). As LRTS follows a chi-square distribution, X was considered equivalent to the best at the 95% level when LRTS < χ2_0.05,1_. Thus, likelihood-based 95% confidence intervals for each parameter were obtained by raising or lowering its value until L was reduced to the value of log(L) = log(L_best_) - χ2_0.05,1_/2 [7].

Softwares

An extensively tested [1,2,4,14-16] single-cycle simulator is the core of modelling cell flow in each of the generations included in the new framework program used in the present study. This program not only renders proliferation through several generations, but simulates a variety of experimental data, including TL, with several dose levels at one time. In a standard PC, typical computational times of a simulation are less than one second.

For non-linear fitting purposes, we implemented the calculation of the objective function and constraints and initially used the Excel Add-in Microsoft Solver®, which is based on the Generalized Reduced Gradient (GRG2) algorithm.

To evaluate the convergence to a unique best-fit scenario in single- and multi-dose models, we repeated the fitting with several randomly chosen starting values of the variables. For these complex and time-consuming fitting purposes, the simulator was implemented also in Matlab (R2012a, Mathworks), both simulators producing the same results. Constrained linear fitting in Matlab was done with the fmincon routine with random multistart. The requirement of multiple (typically 100) repetitions of the same optimization procedure with different randomly chosen starting points was fulfilled by distributing jobs into different computers of a public computer network, running several fits at the same time. The Matlab simulator was compiled as a stand-alone application to run in the Grid platform of the Italian IGI project, which is part of the European Grid Infrastructure (http://web.eu-egi.eu).

The program used to visualize cell cycle distributions of cells (Supplementary Videos 1-5) was made in Matlab.

All softwares are freely available on request from the corresponding author, for non-commercial uses.

In addition, we made three softwares (named PaoSim_Desync, PaoSim_SingleDose, PaoSim_MultiDose ) directly available to the readers as standalone Matlab executable files (downloading them at <https://www.box.com/s/7wq0yhfz761v4e20nlfw> ) with examples of desynchronization routine, of single-dose model and of multi-dose model. Operation of each program is made through the homonymous excel files (supporting Datasets S1, S2 and S3, respectively), which include: 1) a “readme” sheet, with instructions for downloading and running the programs; 2) a “start” sheet with the user interface for input of parameters values; 3) a “Results” sheet where the results of the simulation are given; 4) additional sheets with best fit parameters; 5) datasets for all doses (in Dataset S3).

References

1. Sena G, Onado C, Cappella P, Montalenti F, Ubezio P (1999) Measuring the complexity of cell cycle arrest and killing of drugs: kinetics of phase-specific effects induced by taxol. Cytometry 37: 113-124.

2. Lupi M, Matera G, Branduardi D, D'Incalci M, Ubezio P (2004) Cytostatic and cytotoxic effects of topotecan decoded by a novel mathematical simulation approach. Cancer Res 64: 2825-2832.

3. Ubezio P (2004) Unraveling the complexity of cell cycle effects of anticancer drugs in cell populations. Discrete and Continuous Dynamical Systems - Series B (DCDS-B) 4: 323 - 335.

4. Lupi M, Matera G, Natoli C, Colombo V, Ubezio P (2007) The contribution of p53 in the dynamics of cell cycle response to DNA damage interpreted by a mathematical model. Cell Cycle 6: 943-950.

5. Ubezio P, Lupi M, Branduardi D, Cappella P, Cavallini E, et al. (2009) Quantitative assessment of the complex dynamics of G1, S, and G2-M checkpoint activities. Cancer Res 69: 5234-5240.

6. Begg AC, McNally NJ, Shrieve DC, Karcher H (1985) A method to measure the duration of DNA synthesis and the potential doubling time from a single sample. Cytometry 6: 620-626.

7. Aitkin M, Anderson, D, Freancis, B, Hinde, J (1989) Statistical Modelling in GLIM: Oxford Science Publications.

8. Bevington P, Robinson D (2003) Data reduction and error analysis for the physical sciences. Boston, MA: McGraw-Hill.

9. Motulsky HJ, Christopoulos A (2003) Fitting models to biological data using linear and nonlinear regression. A practical guide to curve fitting.: GraphPad Software Inc.

10. Motulsky HJ, Ransnas LA (1987) Fitting curves to data using nonlinear regression: a practical and nonmathematical review. FASEB J 1: 365-374.

11. Balser JR, Roden DM, Bennett PB (1990) Global parameter optimization for cardiac potassium channel gating models. Biophys J 57: 433-444.

12. Christopoulos A (1998) Assessing the distribution of parameters in models of ligand-receptor interaction: to log or not to log. Trends Pharmacol Sci 19: 351-357.

13. Kleckner IR, Foster MP (2012) GUARDD: user-friendly MATLAB software for rigorous analysis of CPMG RD NMR data. J Biomol NMR 52: 11-22.

14. Lupi M, Cappella P, Matera G, Natoli C, Ubezio P (2006) Interpreting cell cycle effects of drugs: the case of melphalan. Cancer Chemother Pharmacol 57: 443-457.

15. Montalenti F, Sena G, Cappella P, Ubezio P (1998) Simulating cancer-cell kinetics after drug treatment: Application to cisplatin on ovarian carcinoma. Physical Review E 57: 5877-5887.

16. Ubezio P (1990) Cell cycle simulation for flow cytometry. Comput Methods Programs Biomed 31: 255-266.
