# Supplementary material for: Dynamic Rendering of the Heterogeneous Cell Response to Anticancer Treatments
Source: PLoS Comput Biol. 2013 Oct 17;9(10):e1003293. doi: 10.1371/journal.pcbi.1003293 (PMC3798276; doi:10.1371/journal.pcbi.1003293)
Supplement: Text S5 — Antiproliferative response to X-ray exposure. (DOC) [file pcbi.1003293.s019.doc]

**Dynamic rendering of the heterogeneous cell response to anticancer treatments**

F. Falcetta, M. Lupi, V. Colombo and P. Ubezio

**Supporting Text S5: The antiproliferative responses to X-ray exposure**

Rendering the proliferation after X-ray exposure with the best fit of the final model provided a deeper view of the antiproliferative responses to the radiation, with a number of findings that can be compared with published radiobiological studies, and others that are new. Moreover, our study covers a wide range of doses, providing a dose-response of each effect, which was never attempted before to our knowledge.

Probably the most complete published TL studies after X-ray exposure are those of Forrester at al. on rat embryo cells [1] and malignant lymphoid cells [2]. These authors detected different fates of irradiated cells and observed that 1) different death modalities are exploited, such as rapid-interphase apoptosis, delayed apoptosis, and post mitotic apoptosis; 2) cell death may also occur after one or more divisions and is not immediately followed by membrane disruption; 3) cytostatic effects can be long-lasting, part of the cells remaining alive without dividing up to the end of the observation period; 4) polyploidization was important and occurred by re-fusion of sister cells. These effects were also observed in our experiments with IGROV-1 cells, suggesting that they are not peculiar to a specific cell line.

Perturbations of all cell cycle phases have also been reported (see [3,4] for review), using DNA or DNA-BrdU FC analyses, although rarely all the perturbations have been examined at the same time.

Higashikubo et al., with BrdU pulse-chase experiments, detected only G_2_ delays of CHO cells irradiated in S and G_2_ phases at relatively low doses [5], while at higher doses G_1_ arrest and DNA synthesis delay were observed [6]. G_1_ arrest was observed in other cell lines dependent on p53 status [7-9]. Molecular studies have evidenced two mechanisms of G_1_ arrest that are triggered by ionizing radiation: a short-term p53-independent block relying on immediate ATM-dependent cyclin D degradation and a long-term block with higher and persistent p53 levels [10-14].

The IGROV-1 cells used in the present work have functional p53, and we observed a G_1_ block already at 0.5 Gy not only in gen0 but also in gen1 and gen2, with different features. For gen0, part of the cells exposed to X-rays in G_1_ were immediately intercepted at the G_1_ checkpoint, possibly exploiting the rapid response of the p53-independent block pathways. Blockade affected 20% of the G_1_ cells treated with 0.5 Gy, while with 2.5 Gy about 65% of the cells in G_1_ were intercepted by this checkpoint or died rapidly (15%). Because these fractions did not increase at higher doses, the minority of cells (20%) that were not intercepted by the G_1_ checkpoint even at high doses were possibly the closest to the start of S phase and entered S before activation of the block, in keeping with the different radiosensitivity of G_1_-early and G_1_-late cells reported by Yount *et al.* [13]. The G_1_ death rate remained unchanged in the 2.5-10 Gy dose range. A possible threshold for cell kill is therefore between 0.5, where no death was observed, and 2.5 Gy. The main effect on G_1_ of the dose increase between 2.5 and 10 Gy was a reduction of repair, reflected by the recycling rate, which approximately halved when the dose doubled. Recycling was not observed until about 9h after exposure, indicating that the overall process of damage recognition, arrest, processing and release of the block required at least that time, which then lengthened with the dose. Thus, the dose-dependence of G_1_ checkpoint activity in gen0 has two distinct regions: between 0.5 to 2.5 Gy where the blocking activity rises to a maximum of blocked cells, and a second region above 2.5 Gy where the blocking activity is stable but the ability to repair the damage decreases, shifting the death/repair balance towards death.

At the end, among cells irradiated in G_1_, two distinct subsets continued cycling: those that completely bypassed the checkpoint (~75% at 0.5 Gy and ~20% at ≥2.5 Gy) and cells able to recycle after an arrest (from ~40% at 2.5 Gy to 20% at 10 Gy) (regardless of whether they had successfully completed damage repair or not).

For the G_1_ checkpoint activity in the descendants of irradiated cells (gen1 and gen2), with 0.5 Gy it was similar to gen0, while at higher doses it had distinct features. This block affected about half the cells reaching G_1_ with 2.5 Gy, both in gen1 and gen2. Again, the effect reached a plateau at 2.5 Gy, with no increase at higher doses. Recycling was much lower at this checkpoint than for gen0, almost undetectable from 5 Gy, the fate of most blocked cells being death.

For the G_2_M checkpoint, Eriksson demonstrated a transient G_2_ arrest, not enough to complete repair [15]. Alteration of cyclin B-based control led to G_2_ arrest, favouring repair which, however, is not necessarily completed before block release and cell division [16]. Xu et al. suggested two distinct G_2_ checkpoints are activated after X-ray exposure: one short-term, ATM-dependent and dose-independent above 1 Gy, the other long-term, ATM-independent and dose-dependent [17]. ATM in fact is primarily involved in the early response after irradiation in all cell cycle phases [18].

We found that the blocking activity of the G_2_M checkpoint was dose-dependent, very weak with 0.5 Gy but steadily increasing, with more than 98% arrested cells at 10 Gy. More strikingly, the death/repair balance was completely on the side of repair with no detectable death even at the highest dose. Additionally, we could distinguish a gen0 G_2_M block of BrdU^-^ from BrdU^+^ cells. BrdU^+^ cells (irradiated in S) were intercepted by the G_2_M checkpoint but recycled slowly, after a long time in the G_2_M block; both block and recycling showed dose-dependence, with almost all cells blocked and a very slow recycle with 10 Gy.

In the BrdU^-^ subset, we further distinguished cells irradiated in G_2_M, dividing in less than 5h in controls, and cells irradiated in G_1_. The block of BrdU^-^ cells irradiated in G_2_M was weaker than that of BrdU^+^, almost undetectable at 0.5 Gy and brief, with fast recycling up to 5 Gy. However, most of these cells died in G_1_ in gen1 (50% with 2.5 Gy to 95% with 10 Gy, as predicted by the final model) and the majority of cells that divided the second time died in gen2, with 18% (2.5 Gy) to 0.5% (10 Gy) of them reaching gen3. This suggests the G_2_M checkpoint in gen0 was unable to complete repair, allowing the cells to divide even with DNA damage, eventually leading their descendants to death.

Among cells irradiated in G_1_, those which did not experience any delay in G_1_ reached the G_2_M checkpoint between 10 and 24h (2.5 and 5 Gy) or between 20 and 42h (10 Gy, with a delayed passage in phase S), while cells recycled from G_1_ block arrived later. The G_2_M checkpoint intercepted at least part of the cells not arrested in G_1_, while cells recycled from G_1_ were possibly not arrested in G_2_M, although this was not completely resolved by modelling as the exit of these cells from G_2_M overlapped that of other cells recycling from G_2_M block.

The G_2_M checkpoint of gen1 and gen2 descendants was activated with 2.5 Gy, appearing weaker than in gen0, and was negligible with 0.5 Gy. In gen1, G_2_M-blocked cells were able to recycle up to 5 Gy, while in gen2 only a few were able to recycle also at 2.5 Gy, the others being destined to die. In general, repair ability decreased from gen0 to gen1 to gen2.

S checkpoint activity was modest in gen0. Cells irradiated in S-phase were only slightly delayed in S, suggesting this checkpoint is poorly sensitive to direct DNA damage induced by X-rays, while cells irradiated in G_1_ were delayed when they reached S phase, but only at the highest dose. S checkpoint activity became important in gen1 already with 2.5 Gy. The presence of this effect in descendants and not in cells directly exposed to radiation suggests that gen1 cells carry a different kind of damage resulting from previous incorrect replication or mitosis.

Our data thus point to an unexpected high complexity of the antiproliferative mechanisms, with cell cycle impairments/repair not confined in a single phase, extending over generations. Only weak G_1_ and G_2_M blocks were active with 0.5 Gy, while at 2.5 Gy all checkpoints were in play, although with different intensity. Cell cycle impairments on descendants were different from those of directly irradiated cells (gen0), and with 2.5 or higher doses they were similarly active in all cell cycle phases, eventually leading to cell death. This suggests that different checkpoint pathways were activated in gen1 and gen2 from gen0, to deal with different kinds of damage, resulting from previous incomplete processing by proper checkpoints and/or from secondary damage introduced during S and M phases of cells with not fully repaired DNA [19,20]. An incomplete mitotic process is also indicated by the tetraploidization effect, which was also dose-dependent. These damage production/processing mechanisms on descendants may be not limited to gen1 and gen2, but we could not draw conclusions on subsequent generations, as our observation ended at 72h. Studies at longer times, though not technically straightforward, would answer this question.

References

1. Forrester HB, Vidair CA, Albright N, Ling CC, Dewey WC (1999) Using computerized video time lapse for quantifying cell death of X-irradiated rat embryo cells transfected with c-myc or c-Ha-ras. Cancer Res 59: 931-939.

2. Endlich B, Radford IR, Forrester HB, Dewey WC (2000) Computerized video time-lapse microscopy studies of ionizing radiation-induced rapid-interphase and mitosis-related apoptosis in lymphoid cells. Radiat Res 153: 36-48.

3. Bernhard EJ, Maity A, Muschel RJ, McKenna WG (1995) Effects of ionizing radiation on cell cycle progression. A review. Radiat Environ Biophys 34: 79-83.

4. Wilson GD (2004) Radiation and the cell cycle, revisited. Cancer Metastasis Rev 23: 209-225.

5. Higashikubo R, Ragouzis M, Roti Roti JL (1996) Flow cytometric BrdUrd-pulse-chase study of X-ray-induced alterations in cell cycle progression. Cell Prolif 29: 43-57.

6. Bartkowiak D, Hogner S, Nothdurft W, Rottinger EM (2001) Cell cycle and growth response of CHO cells to X-irradiation: threshold-free repair at low doses. Int J Radiat Oncol Biol Phys 50: 221-227.

7. O'Connor PM, Jackman J, Jondle D, Bhatia K, Magrath I, et al. (1993) Role of the p53 tumor suppressor gene in cell cycle arrest and radiosensitivity of Burkitt's lymphoma cell lines. Cancer Res 53: 4776-4780.

8. Kuerbitz SJ, Plunkett BS, Walsh WV, Kastan MB (1992) Wild-type p53 is a cell cycle checkpoint determinant following irradiation. Proc Natl Acad Sci U S A 89: 7491-7495.

9. Basse B, Joseph WR, Marshall ES, Baguley BC (2010) Analysis of radiation-induced changes to human melanoma cultures using a mathematical model. Cell Prolif 43: 139-146.

10. Agami R, Bernards R (2000) Distinct initiation and maintenance mechanisms cooperate to induce G1 cell cycle arrest in response to DNA damage. Cell 102: 55-66.

11. Cann KL, Hicks GG (2006) Absence of an immediate G1/S checkpoint in primary MEFs following gamma-irradiation identifies a novel checkpoint switch. Cell Cycle 5: 1823-1830.

12. Choo DW, Baek HJ, Motoyama N, Cho KH, Kim HS, et al. (2009) ATM is required for rapid degradation of cyclin D1 in response to gamma-irradiation. Biochem Biophys Res Commun 378: 847-850.

13. Yount GL, Haas-Kogan DA, Vidair CA, Haas M, Dewey WC, et al. (1996) Cell cycle synchrony unmasks the influence of p53 function on radiosensitivity of human glioblastoma cells. Cancer Res 56: 500-506.

14. Zhou T, Chou J, Zhou Y, Simpson DA, Cao F, et al. (2007) Ataxia telangiectasia-mutated dependent DNA damage checkpoint functions regulate gene expression in human fibroblasts. Mol Cancer Res 5: 813-822.

15. Eriksson D, Lofroth PO, Johansson L, Riklund KA, Stigbrand T (2007) Cell cycle disturbances and mitotic catastrophes in HeLa Hep2 cells following 2.5 to 10 Gy of ionizing radiation. Clin Cancer Res 13: 5501s-5508s.

16. Kao GD, McKenna WG, Yen TJ (2001) Detection of repair activity during the DNA damage-induced G2 delay in human cancer cells. Oncogene 20: 3486-3496.

17. Xu B, Kim ST, Lim DS, Kastan MB (2002) Two molecularly distinct G(2)/M checkpoints are induced by ionizing irradiation. Mol Cell Biol 22: 1049-1059.

18. Pandita TK, Lieberman HB, Lim DS, Dhar S, Zheng W, et al. (2000) Ionizing radiation activates the ATM kinase throughout the cell cycle. Oncogene 19: 1386-1391.

19. Deckbar D, Birraux J, Krempler A, Tchouandong L, Beucher A, et al. (2007) Chromosome breakage after G2 checkpoint release. J Cell Biol 176: 749-755.

20. Deckbar D, Stiff T, Koch B, Reis C, Lobrich M, et al. (2010) The limitations of the G1-S checkpoint. Cancer Res 70: 4412-4421.
